# Construction and validation of a novel lysosomal signature for hepatocellular carcinoma prognosis, diagnosis, and therapeutic decision-making

**DOI:** 10.1038/s41598-023-49985-3

**Published:** 2023-12-18

**Authors:** Jianlin Chen, Gan Gao, Yufang He, Yi Zhang, Haixia Wu, Peng Dai, Qingzhu Zheng, Hengbin Huang, Jiamiao Weng, Yue Zheng, Yi Huang

**Affiliations:** 1 Shengli Clinical Medical College, Fujian Medical University, Fujian, 350001 Fuzhou China; 2 Department of Clinical Laboratory, Fujian Provincial Hospital, Fujian, 350001 Fuzhou China; 3 Central Laboratory, Fujian Provincial Hospital, Fujian, 350001 Fuzhou China; 4 Center for Experimental Research in Clinical Medicine, Fujian Provincial Hospital, Fujian, 350001 Fuzhou China; 5 Department of Clinical Laboratory, Liuzhou Hospital, Guangzhou Women and Children’s Medical Center, Liuzhou, 545616 Guangxi China; 6 Department of Anesthesiology, The First People’s Hospital of Foshan, Foshan, 528000 Guangdong China; 7 Department of Clinical Laboratory, Fujian Medical University Union Hospital, Fuzhou, 350001 China; 8 Guangxi Clinical Research Center for Obstetrics and Gynecology, Liuzhou, 545616 Guangxi China

**Keywords:** Cancer, Computational biology and bioinformatics, Genetics, Biomarkers, Oncology

## Abstract

Lysosomes is a well-recognized oncogenic driver and chemoresistance across variable cancer types, and has been associated with tumor invasiveness, metastasis, and poor prognosis. However, the significance of lysosomes in hepatocellular carcinoma (HCC) is not well understood. Lysosomes-related genes (LRGs) were downloaded from Genome Enrichment Analysis (GSEA) databases. Lysosome-related risk score (LRRS), including eight LRGs, was constructed via expression difference analysis (DEGs), univariate and LASSO-penalized Cox regression algorithm based on the TCGA cohort, while the ICGC cohort was obtained for signature validation. Based on GSE149614 Single-cell RNA sequencing data, model gene expression and liver tumor niche were further analyzed. Moreover, the functional enrichments, tumor microenvironment (TME), and genomic variation landscape between LRRS^low^/LRRS^high^ subgroup were systematically investigated. A total of 15 Lysosomes-related differentially expressed genes (DELRGs) in HCC were detected, and then 10 prognosis DELRGs were screened out. Finally, the 8 optimal DELRGs (CLN3, GBA, CTSA, BSG, APLN, SORT1, ANXA2, and LAPTM4B) were selected to construct the LRRS prognosis signature of HCC. LRRS was considered as an independent prognostic factor and was associated with advanced clinicopathological features. LRRS also proved to be a potential marker for HCC diagnosis, especially for early-stage HCC. Then, a nomogram integrating the LRRS and clinical parameters was set up displaying great prognostic predictive performance. Moreover, patients with high LRRS showed higher tumor stemness, higher heterogeneity, and higher genomic alteration status than those in the low LRRS group and enriched in metabolism-related pathways, suggesting its underlying role in the progression and development of liver cancer. Meanwhile, the LRRS can affect the proportion of immunosuppressive cell infiltration, making it a vital immunosuppressive factor in the tumor microenvironment. Additionally, HCC patients with low LRRS were more sensitive to immunotherapy, while patients in the high LRRS group responded better to chemotherapy. Upon single-cell RNA sequencing, CLN3, GBA, and LAPTM4B were found to be specially expressed in hepatocytes, where they promoted cell progression. Finally, RT-qPCR and external datasets confirmed the mRNA expression levels of model genes. This study provided a direct links between LRRS signature and clinical characteristics, tumor microenvironment, and clinical drug-response, highlighting the critical role of lysosome in the development and treatment resistance of liver cancer, providing valuable insights into the prognosis prediction and treatment response of HCC, thereby providing valuable insights into prognostic prediction, early diagnosis, and therapeutic response of HCC.

## Introduction

Liver cancer is supposed to be a common tumor of the alimentary system and ranks 6th and 2nd in malignancy of all tumors and cancer-related mortality, respectively^1^. In particular, HCC accounts for the majority of liver cancers histologically^2^. Approximately nine hundred thousand incidences of HCC annually worldwide, while China occupies 50% of these cases^3^. In recent years, as chemotherapy, embolization, targeted therapy (sorafenib, lenvatinib), and immune checkpoint blockade (ICB) have developed, the survival of patients with advanced (inoperable or metastatic) HCC has improved^4,5^, but overall treatment efficacy remains unsatisfactory. Early detection and diagnosis of hepatocellular carcinoma (HCC) is crucial for treatment and prognosis^1^. It has been reported that the 5-year survival rate for the early phase of HCC is over 70%, whereas that for the late stage is only 5%^6^. However, the lack of specific early HCC biomarkers and superior imaging techniques imposes many challenges on early diagnosis. Although circulating tumor markers have the potential to serve as clinically useful biomarkers for the management of HCC, limitations in detection methods hinder their clinical application^7,8^. Currently, alpha-fetoprotein (AFP) remains the only clinically available biomarker for HCC. However, its clinical applicability is subject to controversy and limitations^9^. Therefore, there is an urgent need for new early diagnostic biomarkers to increase the early detection rate of HCC.

Lysosomes were first discovered in 1955 by Christian de Duve, and the discovery of lysosomes revolutionized the understanding of cellular biology and laid the foundation for the exploration of many human diseases related to lysosomal dysfunction^10^. For a long time, lysosomes have been considered as membrane-bound intracellular organelles for the degradation of cellular components, including proteins, lipids, and nucleic acids^11,12^. In recent years, emerging evidence suggests that lysosomes have a close relationship with tumor development and progression^13–15^ and play major role in tumor drug resistance^16^. Moreover, lysosomes are involved in tumor microenvironment remodeling^17,18^ and cancer cell metastasis, making them an important target for cancer therapy^14,19^. Therefore, targeting lysosomes and related processes has been proposed as a potential strategy for overcoming drug resistance and improving cancer treatment efficacy. Recent studies have suggested that duplication and/or overexpression of lysosome-related genes may lead to hepatocarcinogenesis, progression, and metastasis. For example, the lysosomal-associated protein transmembrane 4 beta (LAPTM4B) gene has been found to be upregulated in HCC tissues, and its expression is associated with poor prognosis and increased tumor aggressiveness^20^. Similarly, abnormally elevated glucosylceramides (GBA) are related to the invasion and poor survival of HCC. The mechanism study further showed that artesunate (ART), an anti HCC drug, achieved its anti-tumor effect through the accumulation of GBA targeted autophagy^21^. Other lysosomal-associated proteins, such as ceroid-lipofuscinosis 3(CLN3) and Cathepsin A (CTSA), have also involved in lysosomal trafficking and may play a role in HCC cell migration and invasion^22–24^. Most of the previous studies, however, have focused on the function of a single gene rather than global changes in the transcriptome of lysosome-related genes (LRGs). Therefore, systemic analysis of the LRGs in HCC will provide novel insights into cancer pathogenesis and reveal new targets for liver cancer prevention or therapies.

In this study, a novel LRRS model was established, which demonstrated stability and accuracy in both the discovery and validation cohorts and could serve as an independent prognostic factor and early diagnosis biomarker for HCC. Besides, the difference between two LRRS subgroup in functional enrichment, TME, genetic mutation landscape, tumor mutation burden (TMB), chemotherapy and immunotherapy response were compared. In combination with the LRRS and other clinical indexes, we also construct a nomogram to predict the probability of survival rate for HCC. Notably, we observed specific expression of CLN3, GBA, and LAPTM4B in Hepatocytes based on single-cell RNA sequencing data, where they promoted cell cycle progression and hypoxia responses. Finally, expression profiles of the LRRS model genes were validated in multiple datasets and cell lines. In summary, our study has expanded the exploration of HCC and provided new clinical insights for accurate diagnosis, prognosis, and treatment of HCC patients.

## Methods

### Data acquisition and pre-processing

LIHC RNA-seq data combined with complete clinical data was downloaded from TCGA (https://portal.gdc.cancer.gov/) (50 normal samples; 365 HCC samples) and ICGC (https://daco.icgc.org/) (202 normal samples; 231 HCC samples). The TCGA-LIHC was functioned as the discovery cohort and ICGC cohort (LIRI-JP) was served as the independent validation cohort in this study. Additionally, the GSE144269 (70 normal samples; 70 HCC samples) and GSE76427(52 adjacent tissues; 115 HCC tissues) datasets were collected from the Gene Expression Omnibus (GEO)^25^ database to validate of the expression patterns of model-related genes. All data were converted and standardized as previously reported^26^. An online tool, HPA (http://www.proteinatlas.org) Online database was applied to evaluate protein level of model-related genes via immunohistochemistry (IHC) staining.

### Development of lysosome-related risk score (LRRS)

The set of human lymphoma-related genes (LRGs) was searched with the keyword “lysosome” in website Gene Set Enrichment Analysis (GSEA) (http://www.gsea-msigdb.org/gsea/downloads.jsp), then 550 LRGs were obtained after removing duplicates (Supplementary Table S1). The differentially expressed genes (DEGs) were defined by Limma algorithm (|log2(FC)|> 1.5 & *p*.adj < 0.05) in discovery cohort. Then, the differentially expressed LRGs (DELRGs) were identified by intersecting DEGs with LRGs. Next, DELRGs with survival prediction (*p* < 0.05) were obtained by univariate Cox regression. Afterward, the LASSO regression analysis was conducted and the LRRS was calculated as the following formula:

$${\text{LRRS}}=\sum \limits_{i}^{n}\left(Coefficientof (i)\times Expressionof gene(i)\right)$$

The coefficient ($i$) and expression of gene ($i$) represented the coefficient obtained from LASSO analysis and the normalized expression value of gene ($i$), respectively. HCC samples were then split into two subgroups: high-LRRS group and low-LRRS group, according to the median value of the LRRS. Then, using R's survival package, survival probabilities were calculated. In addition, R package “stats” (version 3.6.0), “umap” (version 0.2.7.0), and “Rtsne” (version 0.15) were then performed respectively for principal component analysis (PCA), uniform manifold approximation and projection (UMAP) and t-statistic neighborhood embedding (tSNE) to illustrate the distribution of the two risk groups. The “timeROC” package was utilized to evaluate the prediction efficiency.

### The association of LRRS with clinical features and diagnostic evaluation of the LRRS

In TCGA cohort, LRRS values were compared, and survival prognosis were analyzed under different stratification of clinical variables. These were further analyzed in the validation cohort. Then, univariate and multivariate Cox regression analyses were performed. For diagnosis, the LRRS levels in TCGA groups were firstly compared, and the receiver operating characteristic (ROC) curves were graphed to evaluate the diagnostic value of LRRS, especially for early diagnosis of HCC. Moreover, the AUCs of ROC were calculated to compare the diagnostic efficacy of the LRRS vs AFP in diagnosing HCC. Finally, further validation was carried out in the ICGC dataset.

### Establish and evaluate a nomogram

Using the R package "rms", a probabilistic model was constructed to predict 1-, 3-, and 4-year survival in combination with age, gender, tumor grade, tumor stage, and LRRS. Simultaneously, calibration curves were plotted to evaluate the prediction accuracy of the nomogram. According to the C-index, the accuracy between nomogram and other prognostic factors was also assessed^27^. Additionally, the decision curve analysis (DCA) was conducted by the “DCA” package to measure the net clinical benefits of various forecasting models^28^.

### Functional enrichment analyses

As described above, DEGs between the LRRS subgroups were isolated using the same protocol. Then, GO and KEGG analysis was performed by the “clusterProfiler” R package. After that, the GESA analysis was carried out using the Hallmark and C2 KEGG gene sets v7.4, which were used in conjunction with the GSEA software (version 4.1.0), with* p* < 0.05 and a FDR of < 0.25 were considered statistically significant^29^.

### Stemness and Immune landscapes analyses

Stemness analysis was performed according to the previous report^30^. For tumor microenvironment analysis, the “estimate” R package was used to calculate the ImmuneScore and StromalScore in TCGA cohort^1^. The TME score was calculated as described previously^31^. The infiltration abundance of 24 immune cells of each HCC cancer sample was estimated by IMMUNCELL AI algorithm^32^. The numbers of 22 tumor-infiltrating immune cell (TIIC) from each sample were determined by using the package "CIBERSORT" (R)^33^.

### Somatic mutations landscapes analyses

Somatic mutation data in “maf” format were downloaded from TCGA GDC data portal^34^, and waterfall plots were then visualized using the “maftools” package in R. Scores for tumor mutational burden (TMB)^35^ and mutant allele tumor heterogeneity (MATH)^36^ were calculated by the “maftools” package in R.

### Prediction of treatment sensitivity

The tumor immune dysfunction and exclusion (TIDE) was calculated to assess the immunotherapy responses in TCGA and validated in the ICGC cohort, as described previously^37^. The cancer-related chemotherapeutic drug sensitivity was predicted via the Genomics of Drug Sensitivity Database following the previous study^38^.

### Single‑cell RNA sequencing analysis

Single-cell sequencing analysis methods were referenced from previously published study^39^. In short, sequencing data was downloaded from GEO(GSE149614) and processed by the “Seurat V4.0” R package.

### Cell culture and RT-qPCR

Human normal liver cell line (LO2) and human liver cancer cell line (HuH-7, HuH-1, HepG2, PLC, and SK-Hep-1) were purchased from Hongshun Biotechnology Co. LTD (Shanghai, China). LO2 cells were cultured in RPMI-1640 (Procell, Wuhan, China) containing 20% FBS (Procell, Wuhan, China). Liver cancer cell lines were cultured in DMEM (Gibco, CA, USA) with 10%FBS. All cells were cultured in a 5% CO_2_ incubator humidified at 37 ℃. Total RNA was isolated using Steady Pure Quick RNA Extraction Kit (Accurate Biology, AG21023, China) according to the manufacturer’s manual. cDNA was synthesized by MCE RT Master Mix for qPCR II (MCEs, NJ, USA). A GoTaq^®^ qPCR Master Mix (A6001, Promega) was used for qPCR. Primers were synthesized by Shangya Biotechnology (Fuzhou, China). CLN3: forward: 5ʹ-CACTTCCCTGAGTCACGCTC-3ʹ, reverse: 5ʹ-ACGAGGTAGATGCTTGGCAG-3ʹ; GBA: forward: 5ʹ-CGGCCCTGGTTAGTGAAGTA-3ʹ, reverse: 5ʹ-CAGCATGAGTAGGCGGACAT-3ʹ; CTSA: forward: 5ʹ-AAATGCTAGTGAGTCGGAGGA-3ʹ, reverse: 5ʹ-TGTTCAGGAAGCGGGAGAAC-3ʹ; BSG: forward: 5ʹ-GTCTGCAAGTCAGAGTCCGT-3ʹ, reverse: 5ʹ-CACGAAGAACCTGCTCTCGG-3ʹ; APLN: forward: 5ʹ-CATGCCTTTCTGAAGCAGGACT-3ʹ, reverse: 5ʹ-GTGAGAGCTGAATGGACGTGA-3ʹ; SORT1: forward: 5ʹ-TCCGTGTGTCAGAATGGTCG-3ʹ, reverse: 5ʹ-GGCTGTTCCACACACTTGGA-3ʹ; ANXA2: forward: 5ʹ-TCTACTGTTCACGAAATCCTGTG-3ʹ, reverse: 5ʹ-AGTATAGGCTTTGACAGACCCAT-3ʹ; LAPTM4B: forward: 5ʹ-TATTGAGTGCCCTGGCTGAT-3ʹ, reverse: 5ʹ-TGCTTGTACGCTCCGTAAGT-3ʹ. For the relative quantification of genes, GAPDH was used as the internal reference (forward: 5ʹ-GGTGTGAACCATGAGAAGTATGA-3ʹ, reverse: 5ʹ-GAGTCCTTCCACGATACCAAAG-3ʹ) following the 2^−ΔΔCT^ method.

## Result

### Identification of DEGs and DELRGs in HCC

**Figure 1:**
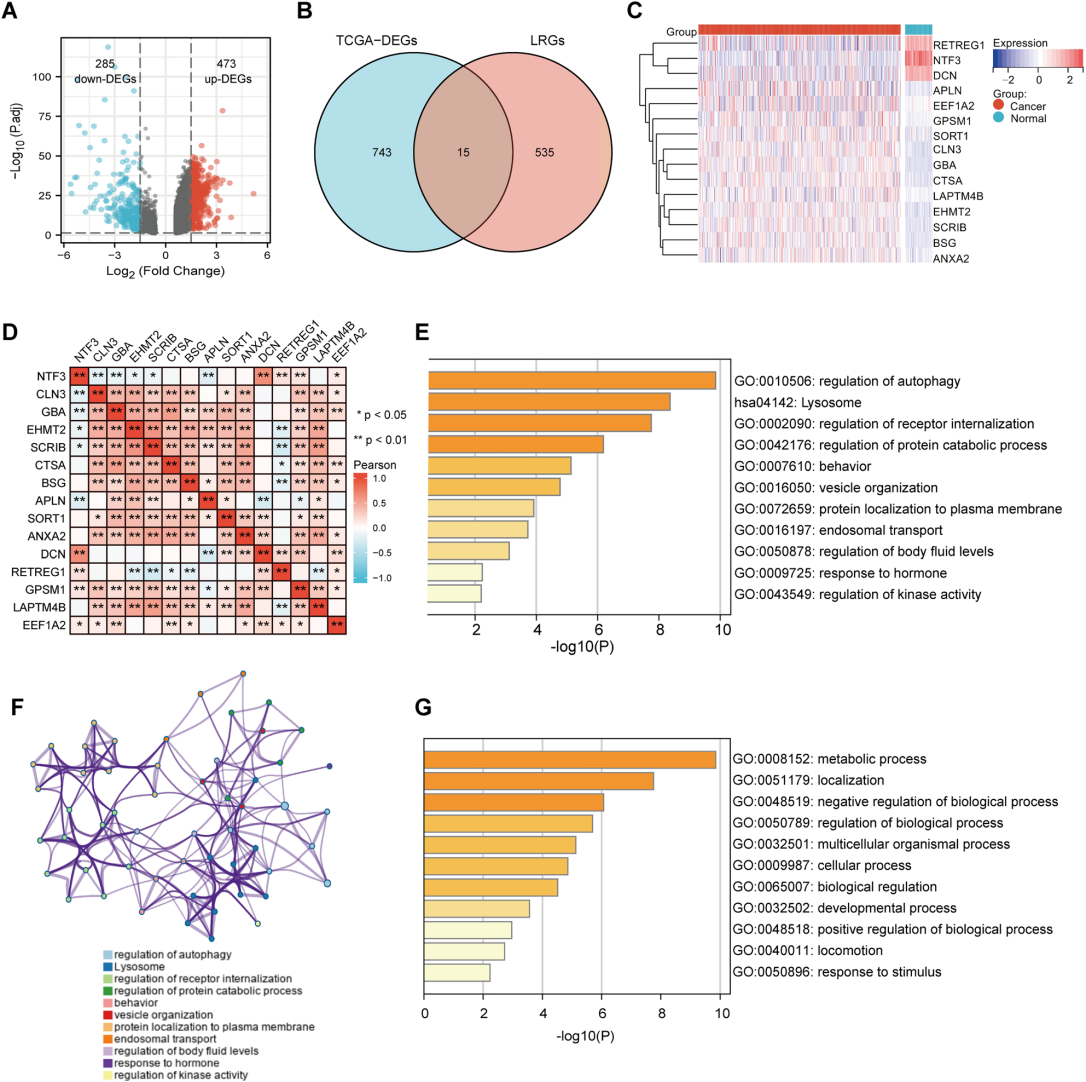
Identification of the lysosomes-related differentially expressed genes (DELRGs). **(A)** Visualization of the differentially expressed genes (DEGs) in TCGA using a volcano plot. **(B)** Overlapping representation of the DEGs and lysosomes-related genes (LRGs) in a Venn diagram. The Venn diagram of the DEGs and LRGs. **(C)** The heat map of 15 DELRGs between HCC and normal tissues in TCGA. **(D)** The Pearson correlation analysis of the DELRGs in TCGA. **(E–G)** The function of the DELRGs in the Metascape database.

There were 758 DEGs (473 upregulated, 285 downregulated) were identified between HCC and normal samples in TCGA (Fig. 1A). The Venn diagram analysis revealed the 15 differentially expressed DELRGs (Fig. 1B). Heatmap showed that 15 DELRGs differed significantly in gene expression between HCC and normal samples (Fig. 1C). As shown in Fig. 1D, DELRGs had a strong correlation. Functional enrichment analyses demonstrated that DELRGs may involve in lysosome and regulation of autophagy (Figs. 1E–G).

### Constructing and validating of the LRRS model

**Figure 2:**
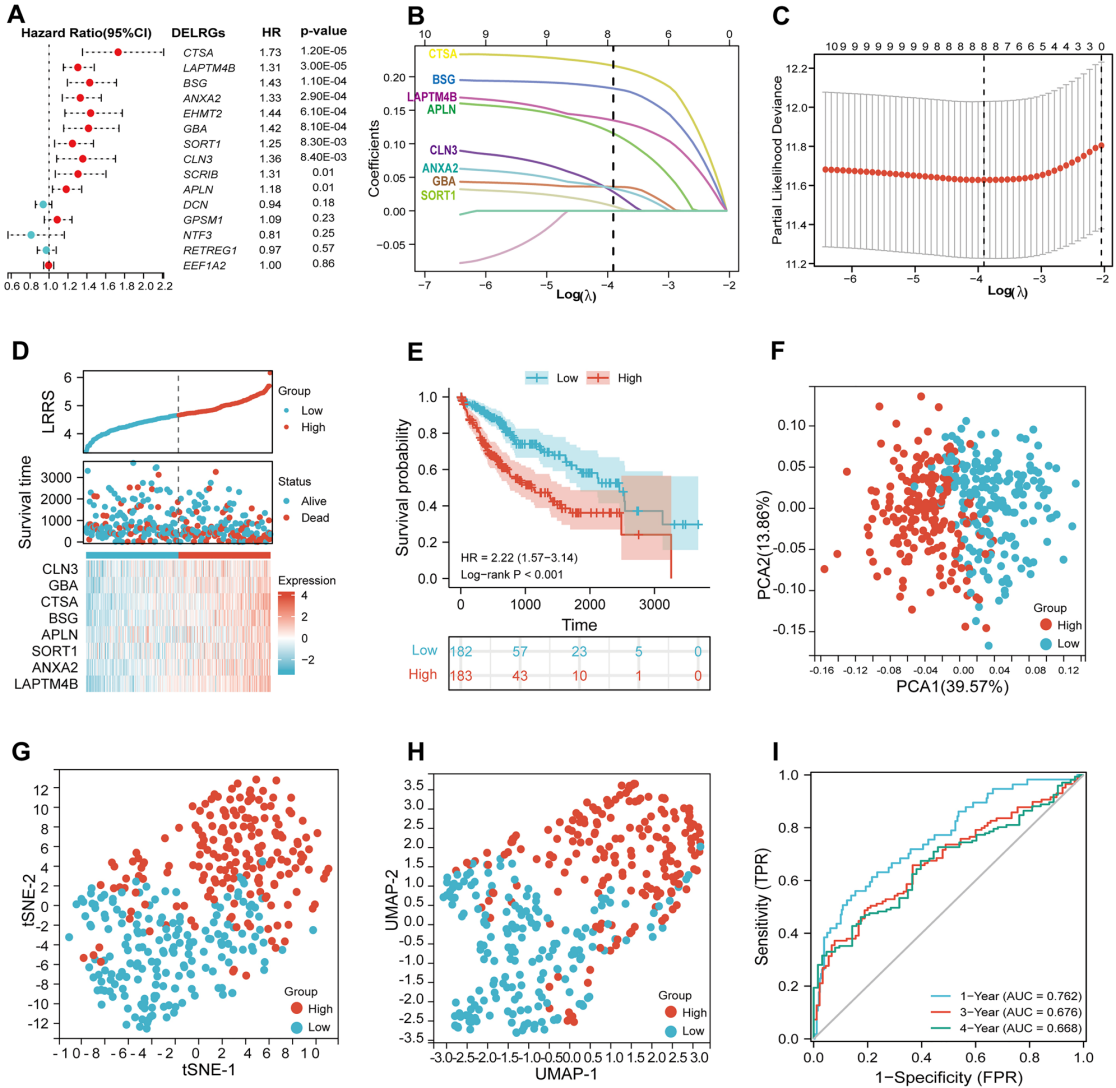
Construction and evaluation of the Lysosome-related risk score (LRRS). **(A)** Univariate Cox regression analysis was performed to assess the predictive value of the 15 DELRGs in the TCGA cohort. **(B)** Construction of the LASSO model. **(C)** The optimal λ value, determining the regularization strength of the LASSO model, was identified for the selected eight LRRS-related genes. **(D)** The risk factor diagram of LRRS model in TCGA cohort was generated, illustrating the significance of the selected genes in predicting risk. **(E)** The overall survival (OS) curve was plotted to compare the outcomes between high- and low-LRRS groups in the TCGA cohort, demonstrating the prognostic value of the LRRS model. Principal component analysis (PCA) **(F)**, t-distributed Stochastic Neighbor Embedding (t-SNE) **(G)**, and Uniform Manifold Approximation and Projection (UMAP) **(H)** were utilized to visualize the LRRS subgroup plot of the LRRS subgroup, portraying its distinct characteristics. **(I)** The 1-, 3-, and 4-year receiver operating characteristic (ROC) curves were constructed to evaluate the performance of the LRRS model in predicting survival outcomes in the TCGA cohort.

Cox regression analysis was shown in Fig. 2A, total of 10 DELRGs (CTSA, LAPTM4B, BSG, ANXA2, EHMT2, GBA, SORT1, CLN3, SCRIB, and APLN) were significantly associated with (Overall Survival, OS), and were selected to construct the LRRS model by LASSO analysis. Then, the optimal eight LRGs model was constructed, with the λ = 0.02(Fig. 2B,C). Following the coefficients, LRRS was calculated by the formula: LRRS = 0.2165 × CTSA + 0.1350 × LAPTM4B + 0.1824 × BSG + 0.0333 × ANXA2 + 0.0353 × GBA + 0.0069 × SORT1 + 0.0279 × CLN3 + 0.1156 × APLN. The distribution of LRRS, survival status, and expression of the model genes in TCGA was presented in Fig. 2D. As can be seen from the PCA, t-SNE, and UMAP analysis, patients were distinctly divided into two groups (Fig. 2F–H). Survival analysis revealed that patients with high LRRS showed shorter survival time (*p* < 0.001, Fig. 2E). The AUCs in time-dependent ROC curves (tROC) displayed decent prognostic accuracy of LRRS for 1-, 2-, and 4-year OS, with the values of 0.762, 0.676, and 0.666, respectively (Fig. 2I).

**Figure 3:**
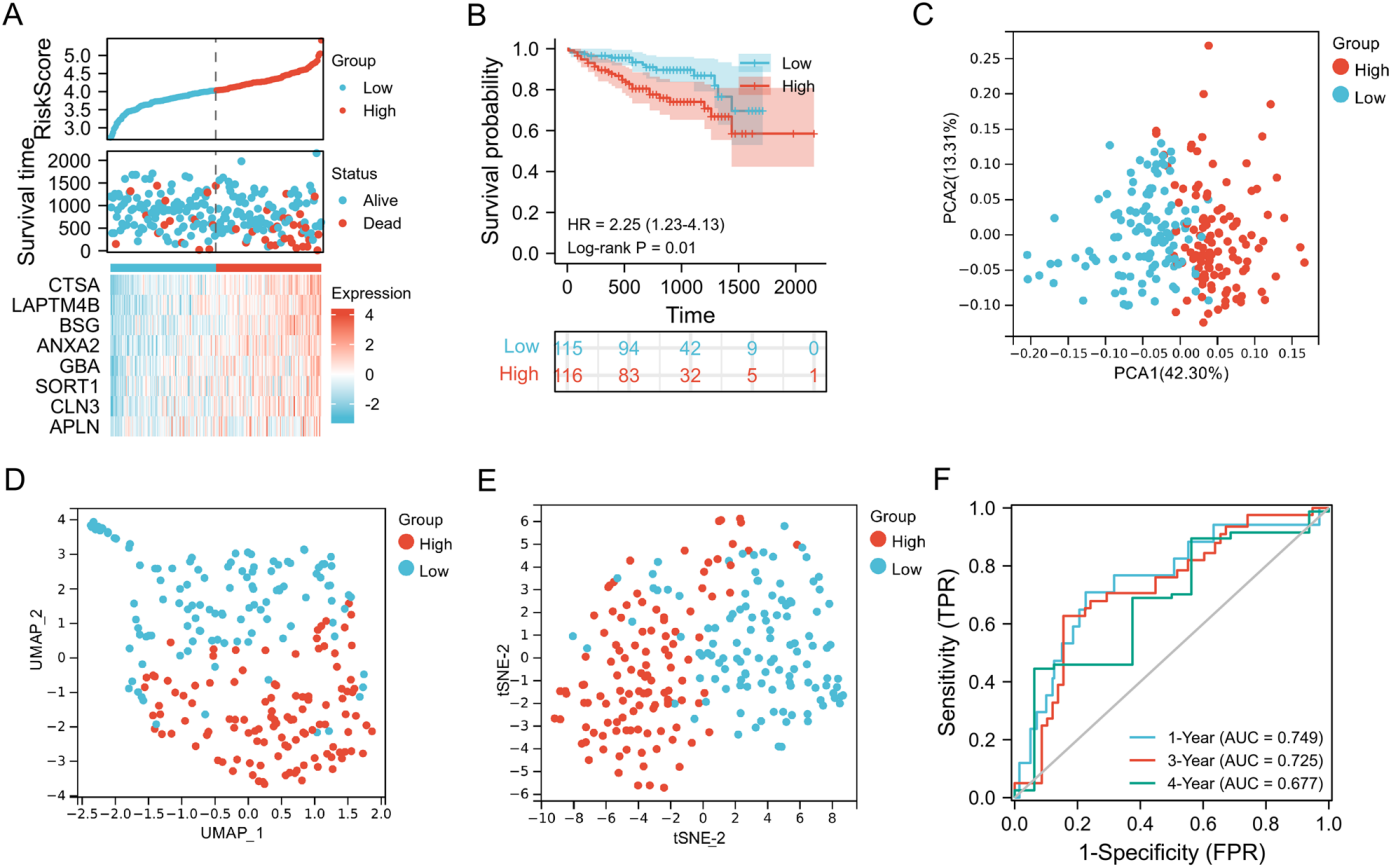
Validation of the LRRS model in the ICGC cohort. **(A)** Risk plot distribution and survival status. **(B)** Kaplan–Meier curves for the OS. **(C)** PCA, **(D)** UMAP, and **(E)** t-SNE plot of the risk model. **(F)** 1-, 3-, and 4-year ROC curves of LRRS model for survival prediction in ICGC cohort.

Later, the prognostic power of the LRRS model was assessed in validation cohort. The LRRS distributions and the expression of model genes within the ICGC cohort can be seen in Fig. 3A. Similar to Fig. 2E, survival analysis in validation cohort (Fig. 3B, *p* = 0.01) completely agrees with the training results. Similarly, Fig. 3C–E illustrated the apparent distribution of subgroups based on LRRS. Moreover, LRRS demonstrated satisfactory prognostic power in validation cohort, with the AUCs for 1-, 3-, and 4-year OS were 0.749, 0.725, 0.677, respectively (Fig. 3F).

### Stratified analysis, independent prognostic analysis, and diagnostic analysis

**Figure 4:**
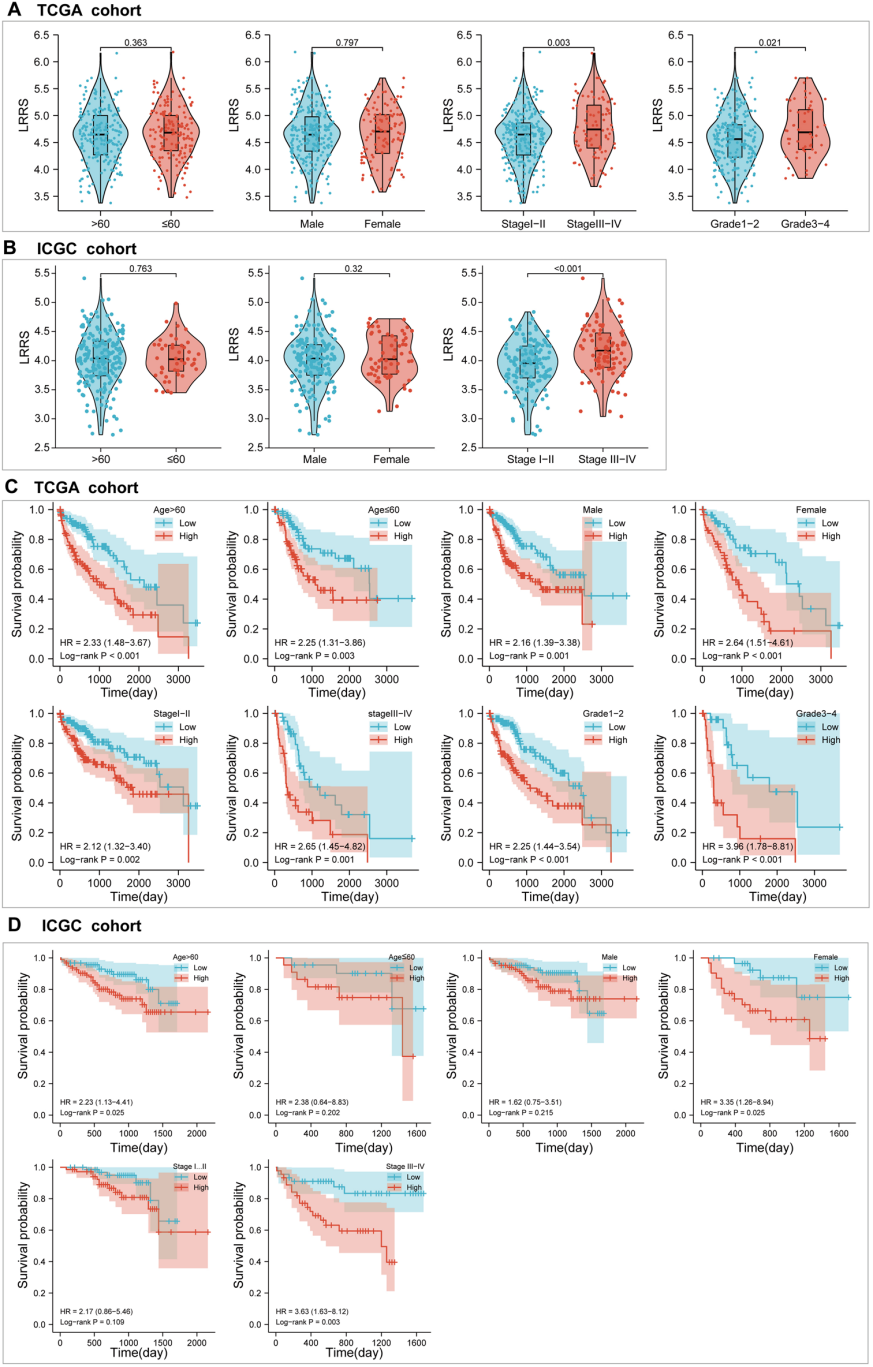
LRRS model-based stratified survival analysis of clinical features in TCGA cohort and validated in ICGC LIRI-JP cohort. Comparison of differences in LRRS between groups based on the clinical parameters of age, gender, stage, and grade using the Wilcoxon signed-rank test in **(A)** TCGA cohort and validated in **(B)** ICGC cohort. Survival analysis of OS stratified by LRRS and HCC clinical parameters in**(C)** TCGA cohort and validated in **(D)** ICGC cohort.

To clarify the correlation between LRRS and clinical features, we further analyzed the differences in LRRS stratified by clinical characteristics in the various subgroups. As shown in Fig. 4A,B, there was no difference in the value of LRRS between the two groups in terms of age (*p* > 0.05) and gender (*p* > 0.05) groups in both training cohort and validation cohort. However, LRRS in advanced stage (III–IV) was apparently higher than that in the early stage (I–II) in both data sets (*p* = 0.003;* p* < 0.001). Likewise, values with LRRS in high grade (3 & 4) was remarkable higher than that for low grade (1 & 2) in TCGA cohort (*p* = 0.021). This demonstrated that the LRRS model had potential correlations with clinical stage and grade of HCC patients. In addition, HCC patients, all in the high LRRS subgroups, presented a poor OS in TCGA cohort (Figs. 4C). In ICGC cohort, patients with high-LRRS also indicated shorter survival time in age (> 60) subgroup, Female subgroup, and stage 3–4 subgroup (Figs. 4D). These findings indicated that our LRRS model maybe a universal applicability tool for prognostic screening.

**Figure 5:**
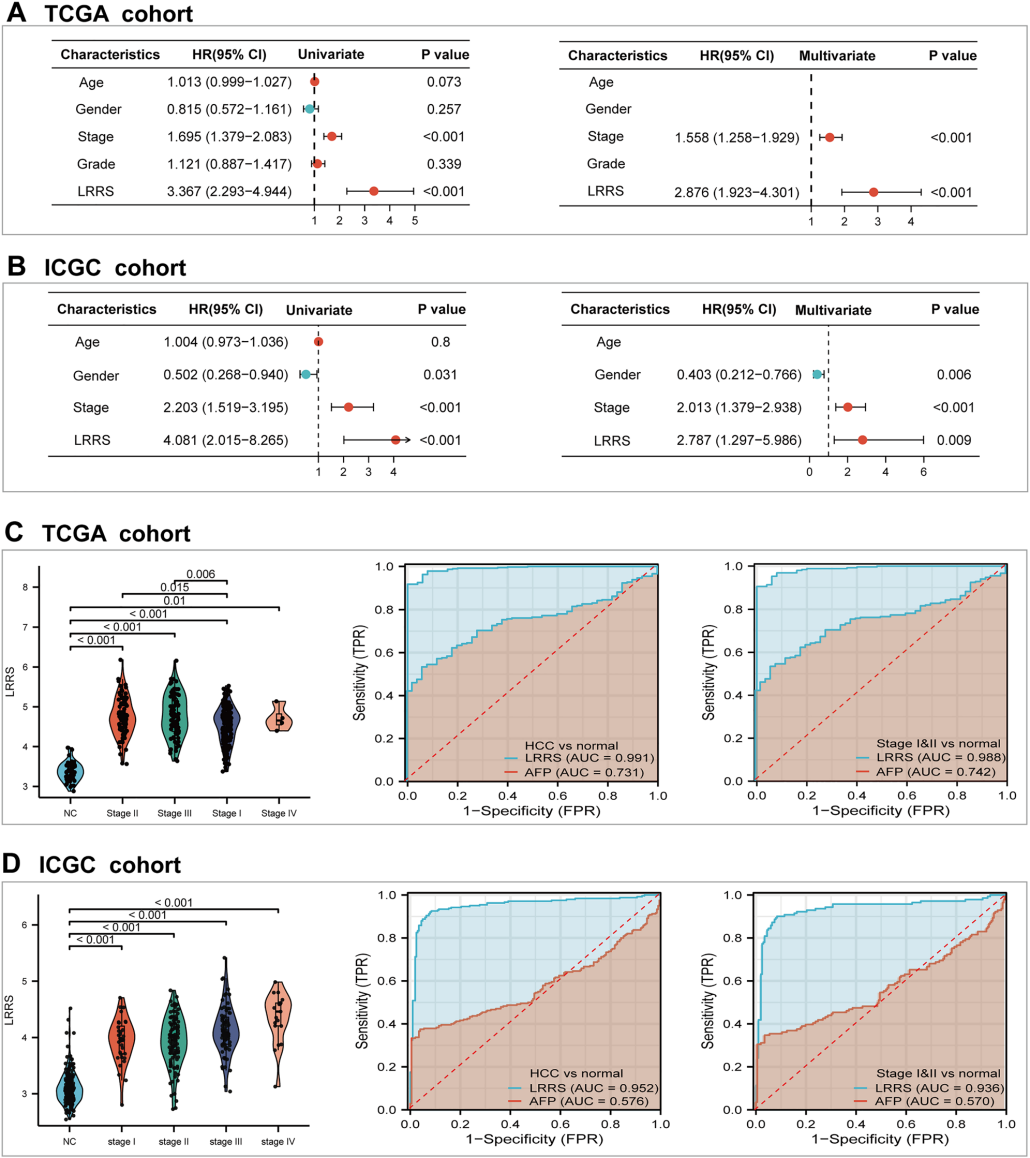
Cox regression analysis and diagnostic analysis of the LRRS signature in HCC. The univariate and multivariate Cox regression analyses in **(A)** TCGA cohort and in **(B)** ICGC cohort. **(C)**. The value of the LRRS in different groups, including normal (n = 50), and HCC tissues at different stages (stages I, n = 177; stages II, n = 88; stages III, n = 86; stages IV, n = 5); ROC curves and AUC values for the LRRS and AFP to distinguish HCC from normal, and to differentiate between normal and patients with early stage (stages I & II) of HCC. **(D)** Diagnostic performance was further validated in ICGC (normal, n = 202; stages I, n = 36; stages II, n = 105; stages III, n = 71; stages IV, n = 19). LRRS levels (mean ± SEM) among multiple groups were statistically analyzed by Ordinary one-way ANOVA.

To verify the ability of the LRRS accurately and independently predict the prognosis of HCC patients, univariate and multivariate Cox regression analyses was performed. In TCGA cohort, univariate Cox regression analysis indicated that the tumor stage (HR = 1.695, 95% CI = 1.379–2.083) and LRRS (HR = 3.367, 95% CI = 2.293–4.944) were relevant risk factors for HCC (both *p* < 0.05). According to multivariate cox regression analysis, stage (HR = 1.558, 95% CI = 1.258–1.929) and LRRS (HR = 2.876, 95% CI = 1.923–4.301) was independent factors for OS (Fig. 5A). In ICGC cohort, stage (HR = 2.203, 95% CI = 1.519–3.195, *p* < 0.001) and LRRS (HR = 4.081, 95% CI = 2.015–8.265, *p* < 0.001) were likewise confirmed to be independent prognostic factors for OS (Fig. 5B).

we further evaluated whether LRRS signature may assist in more accurate diagnosis of HCC. The level of LRRS was found to increase with tumor stage, alluding to a possible novel biomarker for HCC (Fig. 5C, D). Then, the diagnostic performance of the LRRS was evaluated using a ROC analysis. As shown in Fig. 5C, the LRRS vastly outperformed AFP in differentiating HCC from normal samples (AUC: 0.991 vs. 0.731). Likewise, the risk score was superior to AFP in discriminating early-stage HCC patients from normal samples (AUC: 0.988 vs. 0.742). In ICGC validation cohort, the risk score also showed a powerful ability to distinguish HCC from normal (AUC = 0.952) and early-stage HCC from normal (AUC = 0.936) (Fig. 5D). These data suggested that LRRS signature was a potential diagnostic biomarker for HCC, especially for early-stage HCC.

### Development and evaluation of a Nomogram in the TCGA cohort

**Figure 6:**
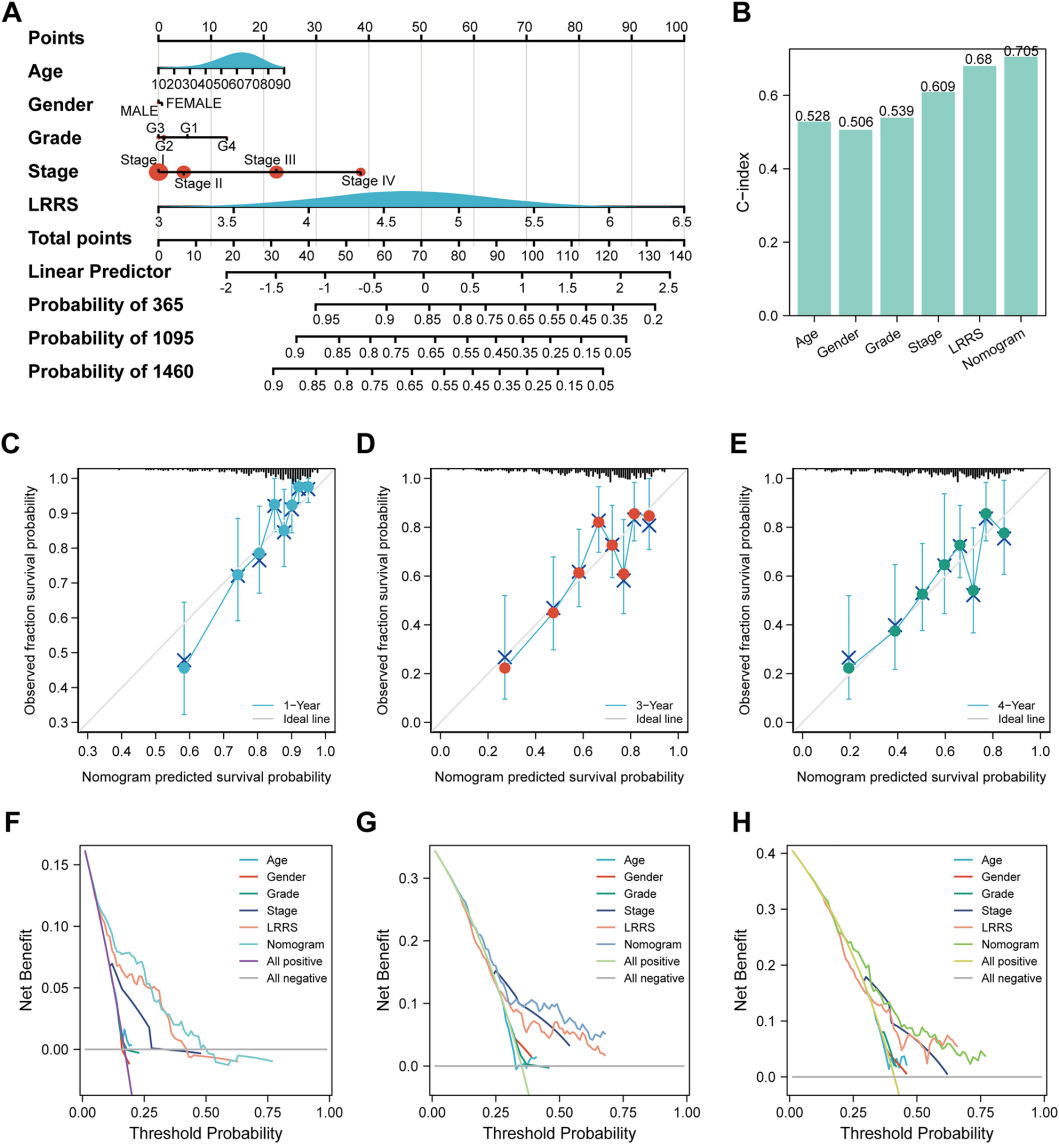
Nomogram to evaluate the OS probability based on TCGA cohort. **(A)** Sophisticated nomogram depicting the estimation of 1-, 3-, and 4-year overall survival probabilities. **(B)** Comparison of C-index among age, gender, grade, stage, LRRS, and nomogram. Calibration curves of the nomogram to predict **(C)** 1-, **(D)** 3- and **(E)** 4-year OS probabilities. Decision curve analysis (DCA) performed to assess the utility of age, gender, grade, stage, LRRS, and the advanced nomogram for **(F)** 1-, **(G)** 2-, and **(H)** 4-year overall survival.

According to the prognostic analysis, a nomogram including LRRS, age, gender, grade, and stage was structured to calculate the OS for HCC (Fig. 6A). The C-index for age, gender, grade, stage, LRRS, and nomogram was 0.528, 0.506, 0.539, 0.609, 0.68, and 0.705, respectively (Fig. 6B). The C-index of nomogram model was more than that of other parameters shown a favorable discrimination ability. From the calibration curves, the 1 -, 3 -, and 4-year observations were in good agreement with the nomogram OS predictions (Fig. 6C–E). The DCA curve also suggested that the nomogram had good clinical assessment than other characteristics (Fig. 6F–H).

### Enrichment analyses

**Figure 7:**
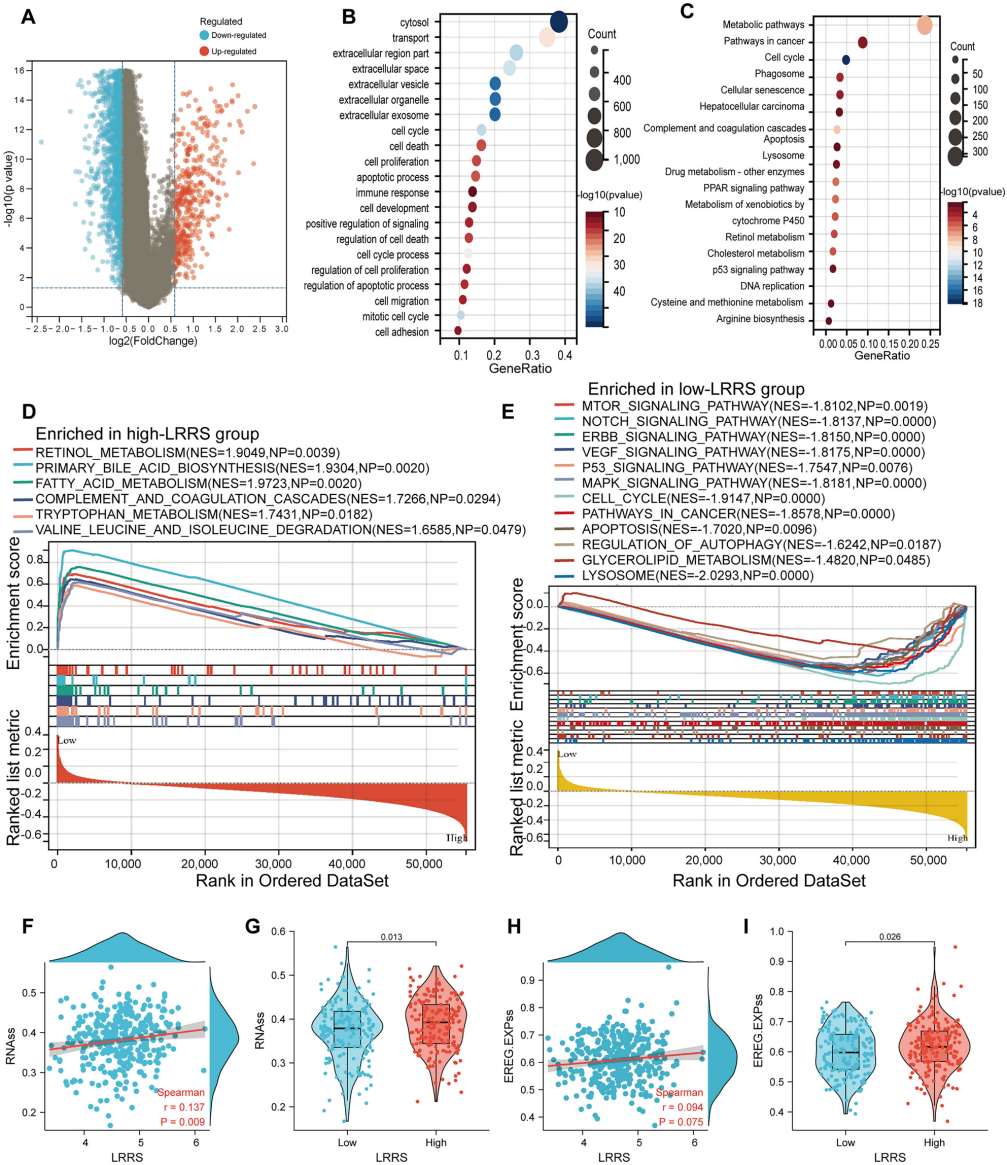
Functional enrichment and stemness analyses between the high and low LRRS groups. **(A)** Displays volcanic map of DEGs observed in the high and low LRRS groups. The GO analysis **(B)** and KEGG analysis **(C)** diagram present the enriched pathways of the DEGs. The GSEA results of for KEGG pathways in the high-LRRS **(D)** and low-LRRS **(E)** groups are illustrated**. (F)** Correlation scatter plot of LRRS and RNAss. **(G)** The violin plot of the difference in RNAss between the high and low LRRS groups. **(H)** Correlation scatter plot of LRRS and EREG.EXPss. **(I)** The violin plot of the difference in EREG.EXPss between the high and low LRRS groups.

By applying the criteria of |log2(FC)|> 1.5 and *p*.adj < 0.05, we have identified a total of 390 up-regulated and 2587 down-regulated DEGs between the high and low LRRS groups, with the low-LRRS group being utilized as the reference. (Fig. 7A). The GO enrichment illustrated that DEGs significant enriched in the vital function of cells, including transport, cell cycle, regulation of cell death, proliferation, apoptotic, migration, cell adhesion (Fig. 7B). Meanwhile, the result of KEGG analysis showed that DEGs were significantly enriched for many metabolic pathways, lysosome, hepatocellular carcinoma, and cancer-related pathways (PPAR signaling pathway and p53 signaling pathway) (Fig. 7C). Similarly, GSEA further confirmed that the high-LRRS group were significantly enriched for various metabolic pathways (retinol metabolism, primary bile acid biosynthesis metabolism of fatty acids, tryptophan metabolism, degradation of valine, leucine, and isoleucine) (Fig. 7D). What is consistent with KEGG analysis is that lysosome, mTOR signaling, Notch signaling pathway, ERBB signaling pathway, VEGF signaling pathway, P53 signaling pathway, MAPK signaling pathway, pathways in cancer, regulation of autophagy and cell cycle were significantly enriched in the low-LRRS group (Fig. 7E).

### Tumor stemness analyses

Studies have reported that tumor cell stemness-related indexes were supposed to be significantly corelated with drug resistance, cancer recurrence and proliferation, and a high index seems to be directly related to the progress of various types of cancer^30,40^. In addition, these index can also help to identify new targets of anti-cancer drugs. Results showed that the value of RNAss and LRRS were positively correlated (Fig. 7F), and the correlation between EREG.EXPss and LRRS was not statistically significant (Fig. 7H). At the same time, parients with high LRRS showed higher values of RNAss (Fig. 7G, *p* = 0.013) and EREG.EXPss (Fig. 7I, *p*  = 0.026) than that of the low-LRRS group.

### Somatic mutations landscapes analyses

**Figure 8:**
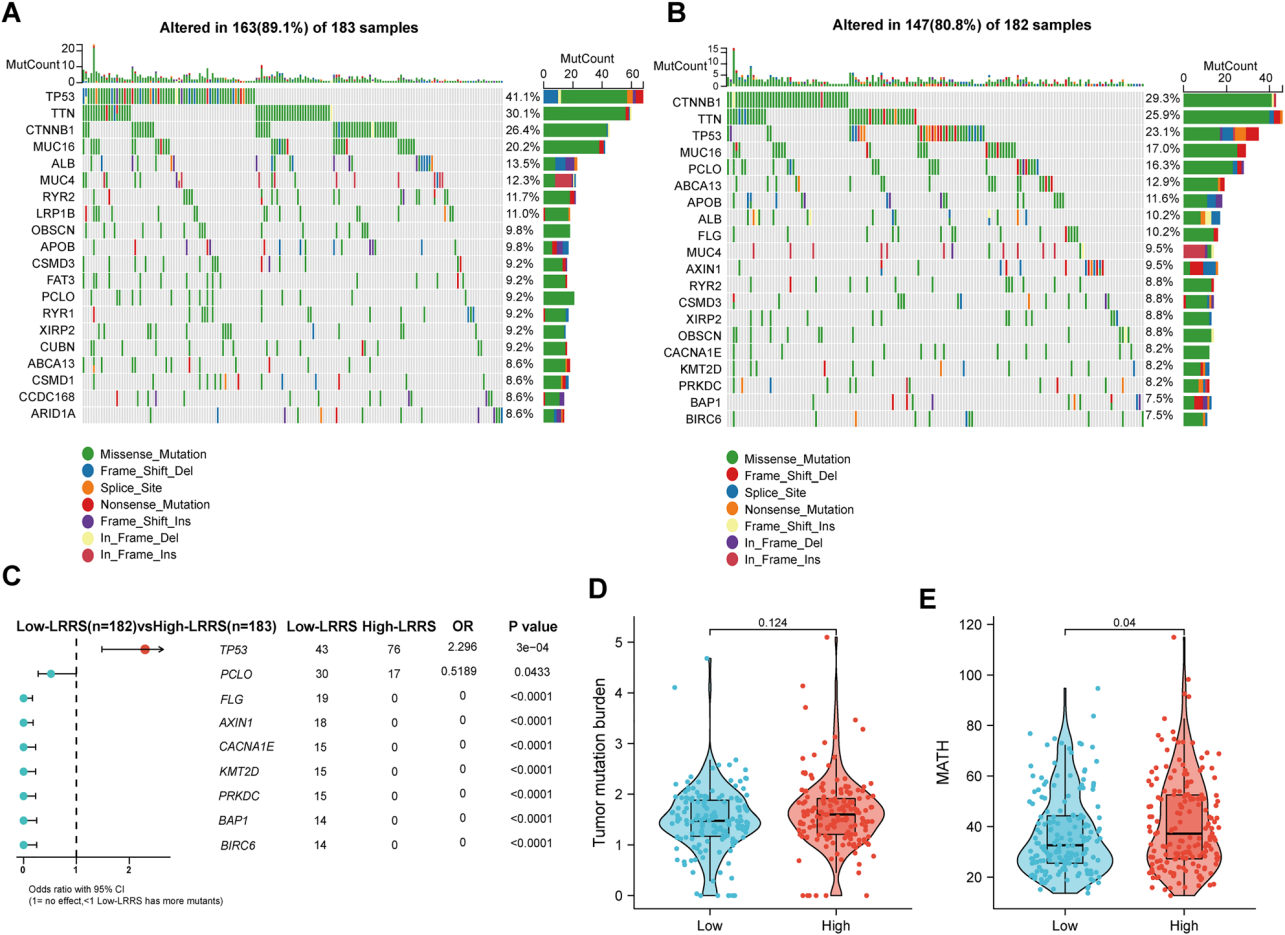
Comparison of somatic mutations between LRRS-based groups. Waterfall maps of mutated genes in HCC patients with high LRRS **(A)** and low LRRS **(B)** groups. **(C)** Forest maps of differentially mutated genes in patients with high LRRS and low LRRS HCC. Comparison of TMB **(D)** and MATH score **(E)** between HCC patients with high and low LRRS. Data were analyzed by Wilcoxon test.

We further analyzed the genomic characteristics of LRRS-based HCC subgroups (Fig. 8A,B). Results presented that the top five mutated genes in the high-LRRS group were TP53 (41.1%), TTN (30.1%), CTNNB1 (26.4%), MUC16 (20.2%), and ALB (13.5%). However, the top five mutated genes in the low-LRRS group were TP53 (23.1%), TTN (25.9%), CTNNB1 (29.3%), MUC16 (17%), and ALB (10.2%). According to Fig. 8C, 43 patients with low-LRRS and 76 patients with high-LRRS were observed to have TP53 mutations (OR = 2.296, *p* < 0.001). Moreover, there was a greater likelihood of finding mutations in PCLO, FLG, AXIN1, CACNA1E, KMT2D, PRKDC, BAP1 and BIRC6 in low-LRRS group (all *p* < 0.0001, Fig. 8C). Surprisingly, although the two groups showed different mutation status, both groups were not statistically different in TMB (Fig. 8D, *p* = 0.124). Interestingly, the MATH score, an indicator of tumor heterogeneity, was found to be significantly higher in patients with high LRRS (Fig. 8E, *p* = 0.04).

### Immune landscapes analyses

**Figure 9:**
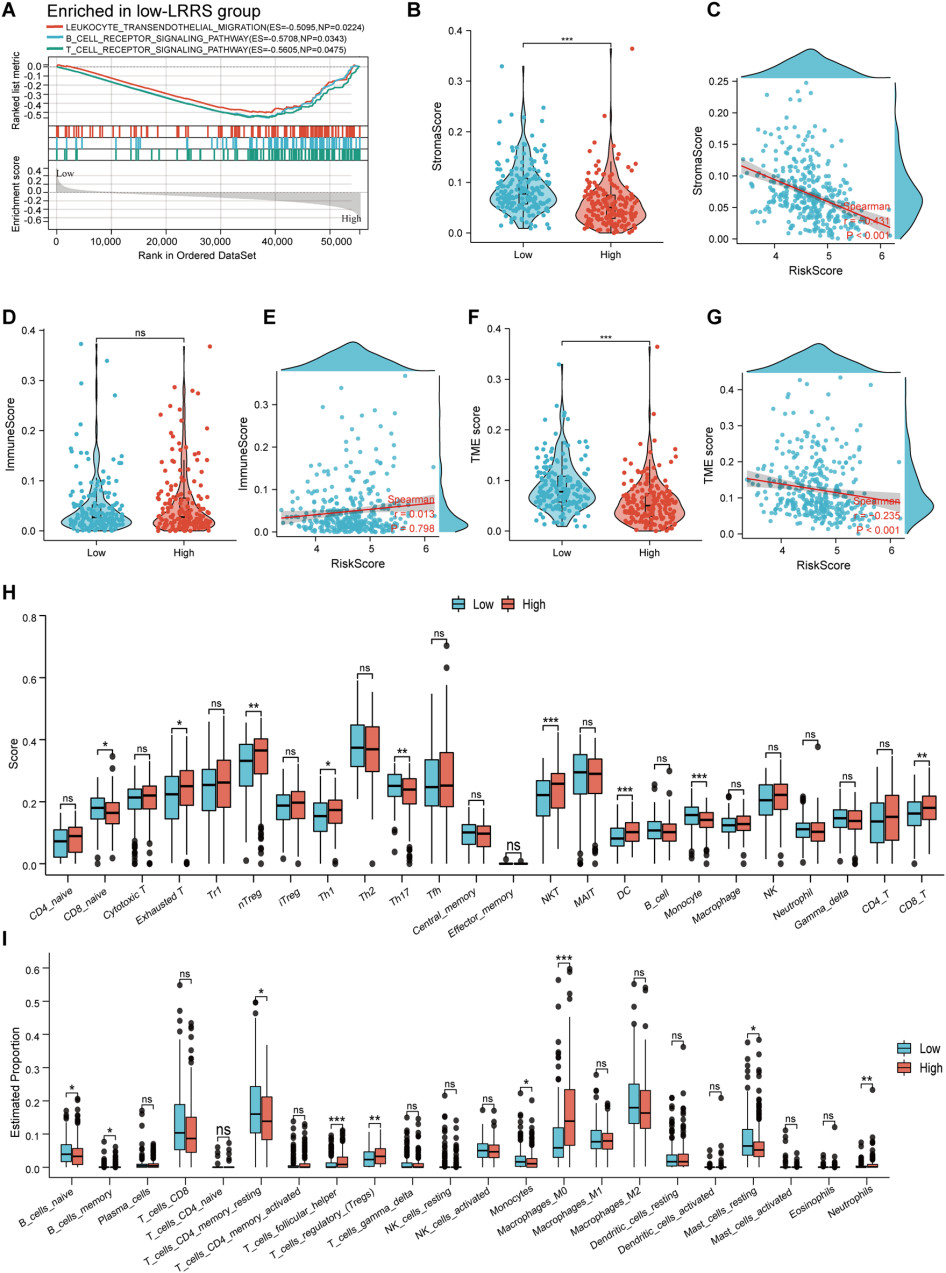
Immune landscapes between the high and low LRRS groups. **(A)** GSEA analysis shows the significant enrichment in immune-associated biological processes in the low-LRRS group. StromaScore **(B)**, ImmuneScore **(D)**, and TME score **(F)** in different LRRS groups, respectively. Spearman correlation analysis of the LRRS and StromaScore **(C)**, ImmuneScore **(E)**, and TME score **(G)**, respectively. The landscape of immune cell infiltration between two LRRS subtypes estimated by the CIBERSORT algorithm **(H)** and ssGESA **(I)**. ns ≥ 0.05, * < 0.05, ** < 0.01, and *** < 0.001.

According to the GSEA analysis, we found immune-related pathways(leukocyte transendothelial migration, B-cell and T-cell receptor signaling pathways) were significantly enriched in the low LRRS group (Fig. 9A). Interestingly, samples in low-LRRS exhibited significantly higher StromalScore, compared with that of high-LRRS group (Fig. 9B, *p* < 0.05), as were the TME score (Fig. 9F, *p* < 0.001).However, both groups were not statistically different in ImmuneScore (Fig. 9D, *p* > 0.05). Furthermore, the correlation of the three scores and LRRS was also explored. The results showed that except for ImmuneScore, both StromalScore and TME score were significantly negatively correlated with LRRS(Fig. 9C, E, G). Then, based on the TCGA cohort,the infiltration level of 24 immune cells were evaluated using ssGSEA by the ImmuneCellAI online tool. Surprisingly, both groups were not statistically different in the infiltration of most immune cells, including, NK cells, CD4+ T cells, neutrophils, B cells,Th2, and cytotoxic cells, while patients in the low-LRRS group showed a higher fraction of anti-tumor immune cells, such as CD8-naive (*p* < 0.05), Th17 cells (*p* < 0.01), and Monocyte (*p* < 0.001) (Fig. 9H). Moreover, a higher level of exhausted T (*p* < 0.05) (a group of T cells that have reduced effector function and continue to express inhibitory receptors), Th1 (*p* < 0.05), NKT (*p* < 0.001), DC (*p* < 0.001), CD8+ T (*p* < 0.01) and nTreg (*p* < 0.01) were observed in the high-LRRS group (Fig. 9H). Furthermore, we used the CIBERSORT algorithm to verify the infiltration level of immune cells in TCGA cohort and found that the low-LRRS group shown a higher infiltration of a variety of anti-tumor immune cells, including B naive cells (*p* < 0.05), CD4+ T memory resting cells (*p* < 0.05), Monocytes (*p* < 0.05), and Mast resting cells (*p* < 0.05), while high-LRRS group showed a higher estimated proportion of tumor-promoting cell, Tregs (*p* < 0.01) (Fig. 9I). At the same time, we found that the high LRRS group showed a high proportion of M0 infiltration (*p* < 0.001) (Fig. 9I). It is well known that the elevation of M0 macrophages may represent some adverse immune response, such as autoimmune diseases or cancer. This is highly consistent with the results of ssGESA. All these data indicated that the LRRS is involved in the regulation of immune microenvironment and may affect the anti-tumor immune response in tumors.

### Role of LRRS in clinical decision-making

**Figure 10:**
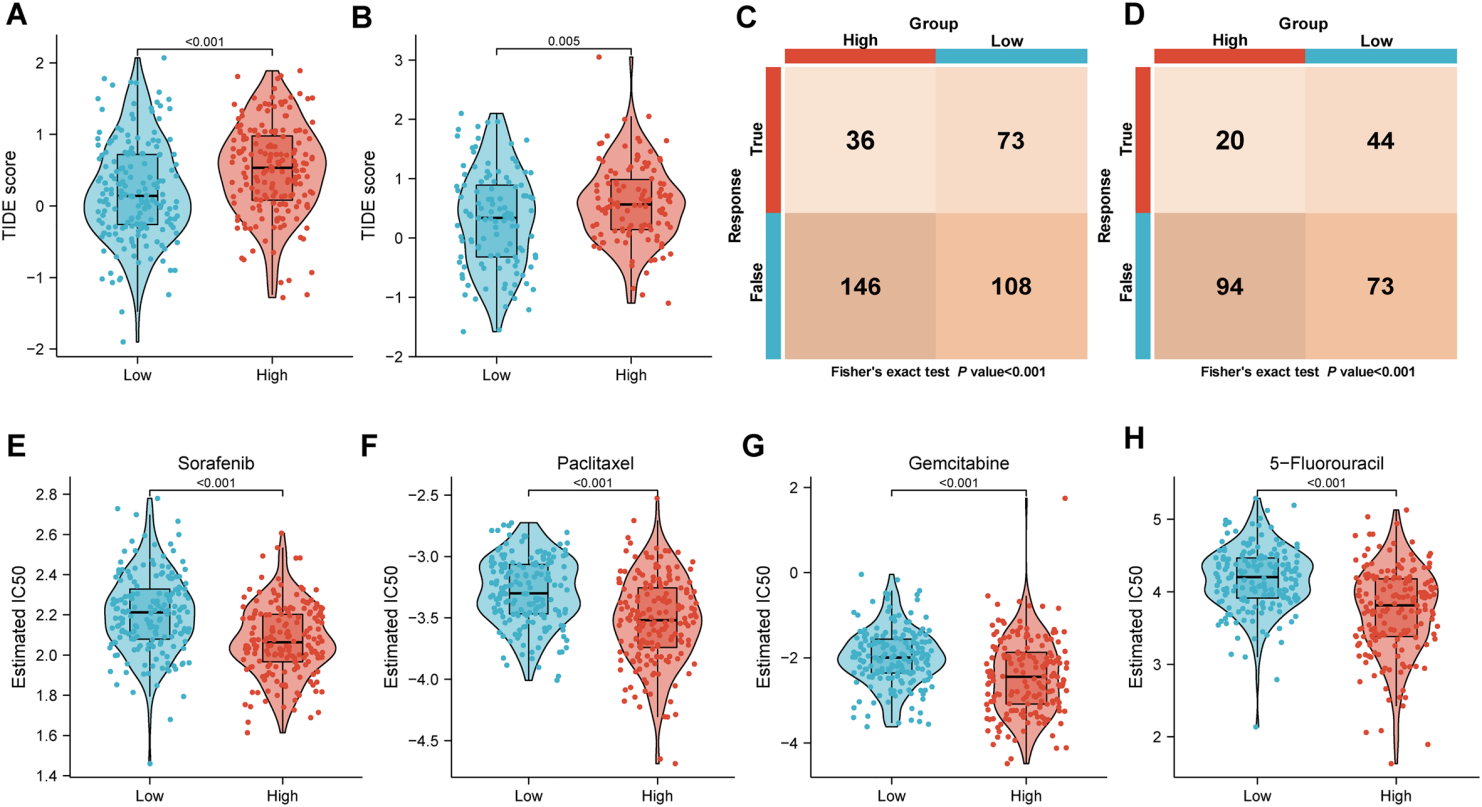
Role of LRRS in clinical decision-making. The TIDE score between two LRRS groups in TCGA cohort **(A)** and ICGC cohort **(B)**. TIDE algorithm to predict the responses of patients in the high- and low-LRRS groups to immunotherapy in TCGA cohort **(C)** and ICGC cohort **(D)**. The chemotherapy response of two LRRS groups for Sorafenib **(E)**, Paclitaxel **(F)**, Gemcitabine **(G)**, and 5-Fluorouracil **(H)** four common chemotherapy drugs.

Given the difference in the TME between the high and low LRRS groups, TIDE algorithm was subsequently applied to predict patients' response to immunotherapy. Previous studies have reported that higher TIDE scores were associated with poorer response to immune checkpoint blocking therapy (ICB) and shorter survival after ICB treatment^37^. Our results showed that the high-LRRS group had a higher TIDE score in the TCGA dataset(Fig. 10A, *p* < 0.001), which was confirmed in the ICGC dataset(Fig. 10B, *p* = 0.005). Furthermore, subclass mapping results indicated that low-LRRS goup showed a more sensitive immunotherapy response in bothTCGA and ICGC cohorts (Fig. 10C, D, all* p* < 0.001). Moreover, based on IC50 values, the sensitivity of four common chemotherapy drugs was further analyzed. we discovered that the high-LRRS group was sensitive to all four drugs (Sorafenib, Paclitaxel, Gemcitabine, and 5-Fluorouracil) (Fig. 10E–H, all* p* < 0.001). In conclusion, these findings indicated that LRRS was is a feasible tool to instruct clinical treatment decisions of HCC patients.

### CLN3, GBA, and LAPTM4B may be novel biomarkers in Hepatocytes

**Figure 11:**
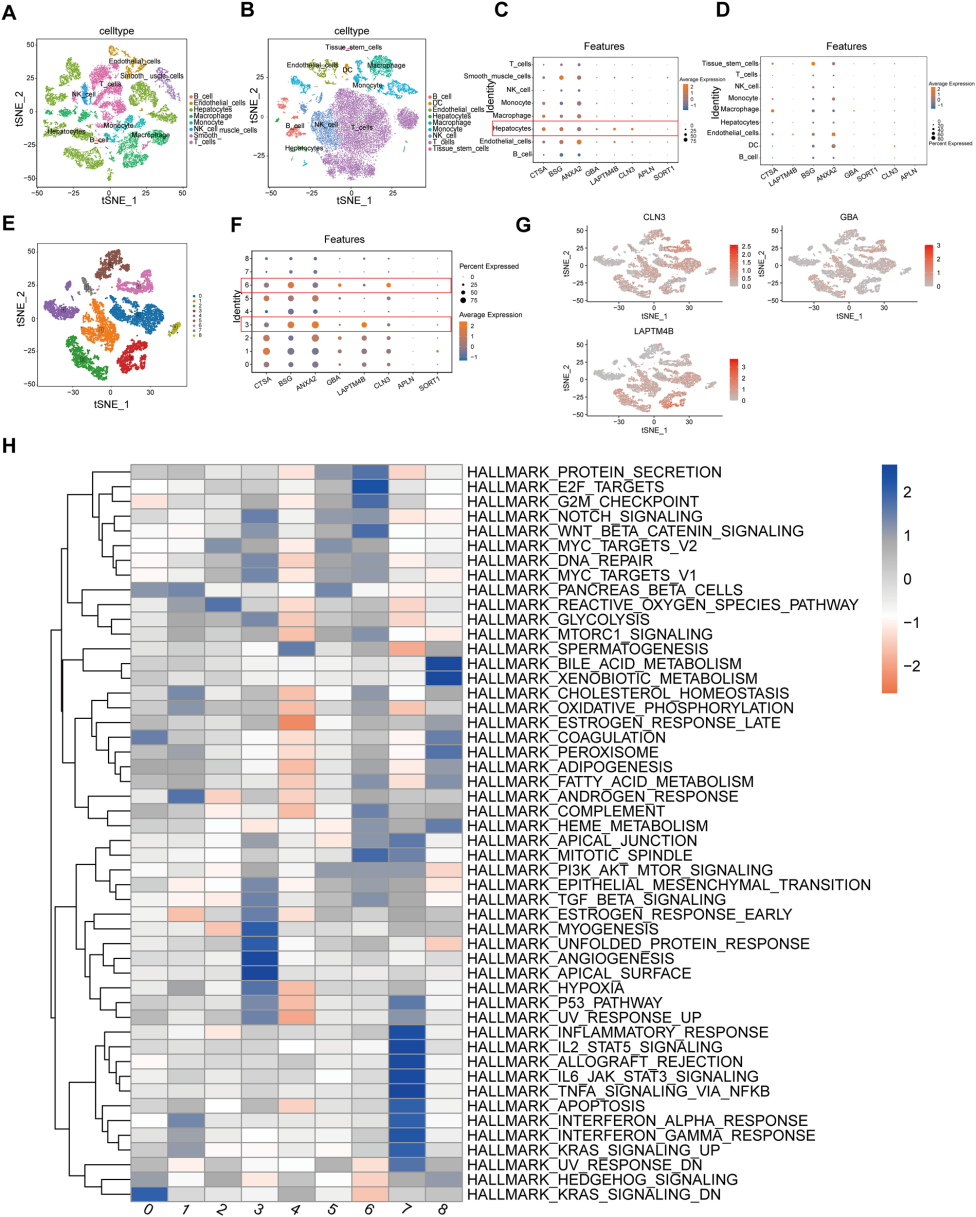
Single cell sequencing analysis in the GSE149614 dataset. (**A,B**) tSNE plot showing predicted cell types in 10 liver tissues **(A)** and 8 paired non-tumor tissues **(B)**. **(C,D)** The expression distribution of the LRRGs signature (CLN3, GBA, CTSA, BSG, APLN, SORT1, ANXA2, and LAPTM4B) in tumor tissues **(C)** and non-tumor tissues **(D)**. **(E)** Sub-clustering of Hepatocytes. **(F)** Dot plot of the LRRGs signature genes in sub-clustering of hepatocytes. **(G)** Feature plot of CLN3, GBA, and LAPTM4B in hepatocytes. **(H)** Heatmap of the KEGG pathways and the HALLMARK gene sets in subsets of hepatocytes.

To further elucidate the special role of LRRS gene signature in HCC progression, single-cell RNA sequencing analysis was performed to investigate the expression profiles of LRRSs in the liver tumor microenvironment. Firstly, 192,675 cells from 10 primary liver tumors and 23,277 cells from 8 non-tumor liver tissues were obtained after quality control filtering. Afterward, these cells were merged, clustered, and annotated. Finally, these cells were mapped to B cells, Endothelial cells, Hepatocytes, Macrophage, Monocyte, NK cells, Smooth muscle cells, Dendritic cells,Tissue stem cells and T cells based on cell-type-specific marker genes (Fig. 11A, B). Remarkedly, the cell types differ greatly amongst tumor and non-tumor tissues (Fig. 11A, B). Subsequently, we mapped the expression landscape of 8 lysosomes-related genes, including CLN3, GBA, CTSA, BSG, APLN, SORT1, ANXA2, and LAPTM4B. As shown in Fig. 11C, D, BSG and ANXA2 were widely expressed in almost all clusters, implying their essential role in cell viability. Meanwhile, CTSA were highly expressed in Macrophage and Hepatocytes. Of note, CLN3, GBA, and LAPTM4B were specially expressed in Hepatocytes, which may be novel biomarkers for liver cancer Hepatocytes(Fig. 11C). Given the high specificity of CLN3, GBA, and LAPTM4B in Hepatocytes, we next elucidated their role in Hepatocytes function. The Hepatocytes were classified into nine subpopulations via dimensionality reduction (Fig. 11E). Notably, CLN3 and GBA were highly expressed in Hepatocytes (6), and LAPTM4B was highly expressed in Hepatocytes (3) (Fig. 11F, G). Furthermore, functional enrichment analysis of Hepatocytes (6)-specific genes revealed strong enrichment of protein secretion, G2M checkpoint, E2F pathways, Wnt/β-catenin signaling and mitotic spindle. Moreover, LCSC (3) strong enrichment of cancer hall markers related to Notch signaling, Glycolysis, PI3K/AKT/mTOR signaling, TGF-βsignaling, Angiogenesis, P53 pathway and Hypoxia (Fig. 11H). Therefore, CLN3, GBA, and LAPTM4B may be involved in cancer progression of liver cancer hepatocytes.

### The expression confirmation of model genes

**Figure 12:**
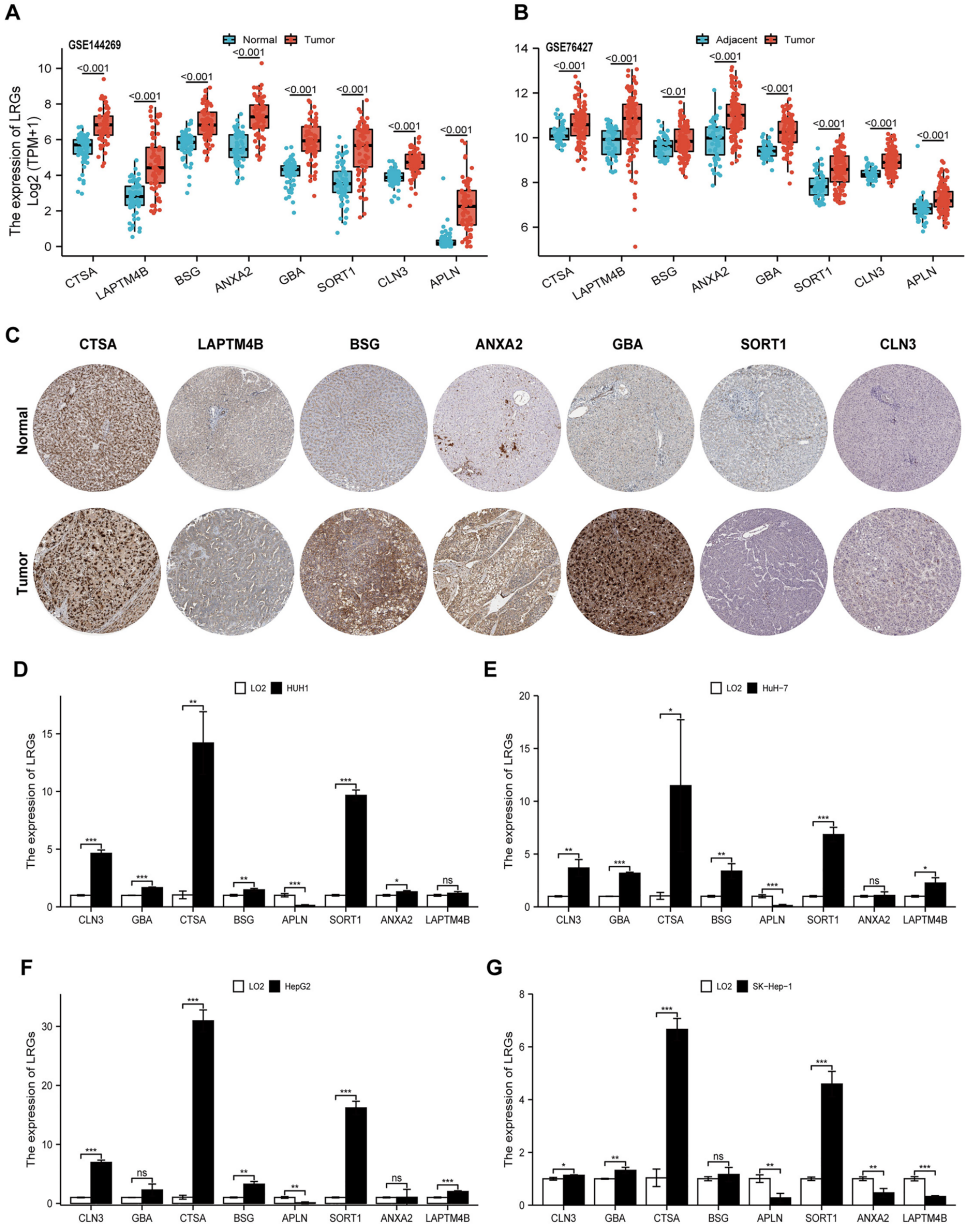
Validation of the expression patterns of 8 signature genes. **(A,B)** The expression levels of eight LRRS model genes in GSE144269 **(A)** and GSE76427 **(B). (C)** HPA database showing the expression of signature gene proteins in HCC tissues compared with normal tissues. **(D–G)** Relative expression of model genes in HuH-1 **(D)**, HuH-7 **(E)**, HepG2 **(F)**, and SK-Hep-1 **(G)** cell lines.

Compared with normals, all eight genes were highly expressed in HCC in GSE144269 (Fig. 12A, all *p* < 0.001), and which was validated using data from the GSE76427 dataset(Fig. 12B, all *p* < 0.001) Subsequently, the protein expression of these genes were analyzed in the HPA database. According to the results, the protein expression of model genes in tumor tissue were elvated (Fig. 12C). Furthermore, the relative expression of model genes were also confirmed in HCC cell lines. Compared with normal cell line(LO2), the model genes were highly expressed in multiple tumor cells (Fig. 12D–G). Overall, these results further validated the stability and reliability of the LRRS model.

## Discussion

**Figure 13:**
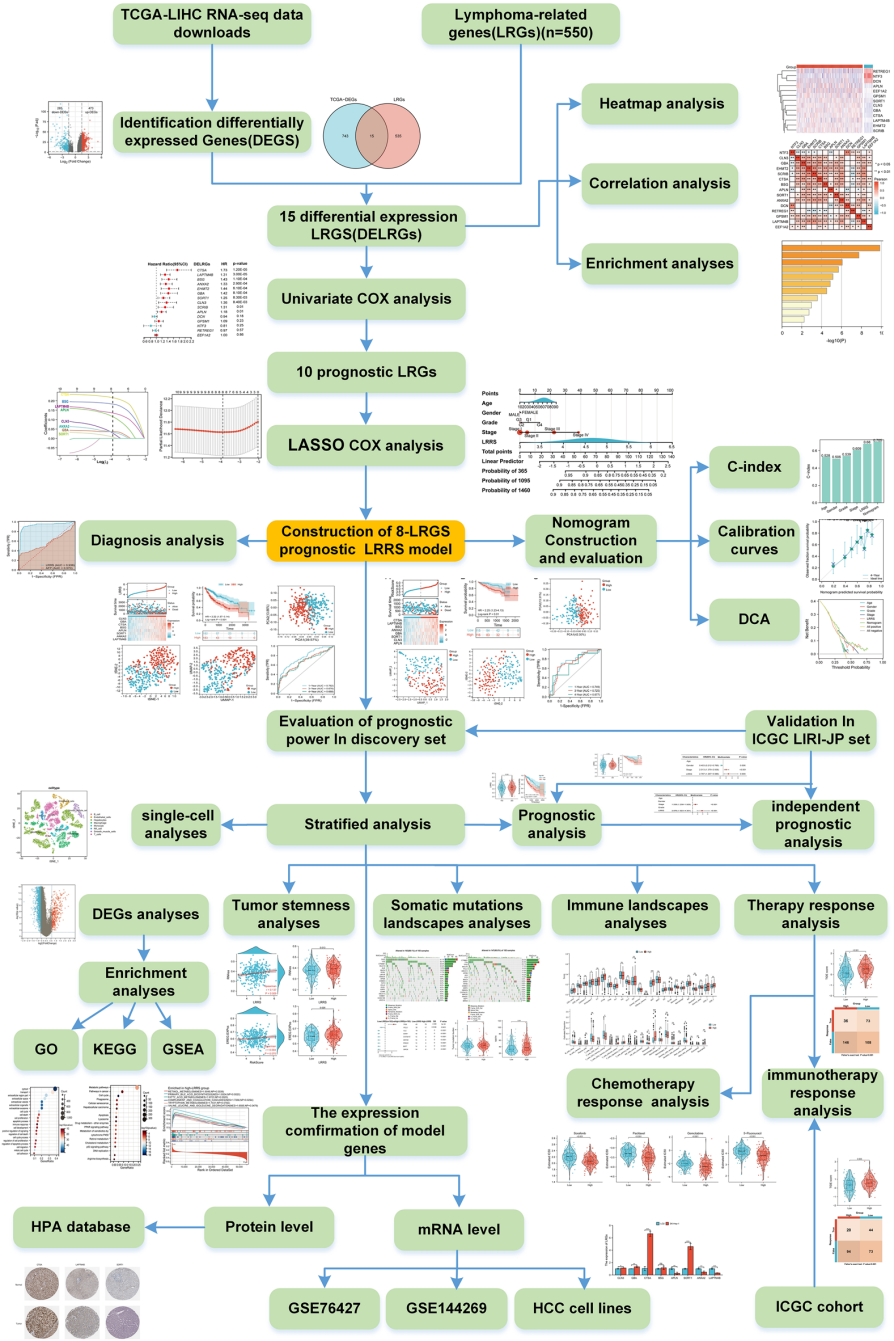
Workflow of this study.

Previous studies have indicated that lysosomal-related genes may serve as potential targets for cancer therapy^14,16,41^. However, the clinical relevance of lysosomal-related genes in the diagnosis and treatment of primary liver cancer has not been fully elucidated. In this study, we demonstrated the key role of lysosomal-related genes (LRGs) in HCC through functional enrichment analysis of differentially expressed genes. Subsequently, we identified ten LRRGs that were overexpressed in HCC and associated with poor prognosis. Furthermore, we constructed a panel of eight LRRGs that exhibited good performance in the diagnosis and prognosis of HCC patients. In summary, the comprehensive transcriptomic analysis of lysosomal-related genes in this study provides insights into the role of lysosomes in HCC (Fig. 13).

Due to the vital role played by lysosomes in cancer, a LRRS signature was constructed, including 8 genes, namely, CLN3, GBA, CTSA, BSG, APLN, SORT1, ANXA2, and LAPTM4B. Combined with literature reports and our analysis, all eight model genes were abnormally high expressed in HCC. Ceroid-lipofuscinosis 3(CLN3), encodes a lysosomal transmembrane protein, which functions as a necessary clearance enzyme for lysosome to clear glycerophosphate diesters (GPDs)^42^. By activating the EGFR/PI3K/AKT pathway, its upregulation leads to tumor growth and metastasis in HCC^22^. Glucosylceramidase (GBA) is considered as a necessary enzyme for autophagic degradation^43^ and has been linked to a variety of cancers in humans^44–46^. A recent study found that abnormally elevated GBA is correlates with HCC invasion and poor survival,which further showed that artesunate(ART), an anti HCC drug, achieved its anti-tumor effect through the accumulation of GBA targeted autophages^21^. According to a previous research, LAPTM4B promotes tumor growth and autophagy in HCC cells by activating ATG3 transcription^20^. More importantly, our study revealed for the first time that CLN3, GBA, and LAPTM4B are specifically expressed in hepatocytes in the liver and promote the progression of liver cancer through multiple tumor-related pathways. This further suggests a potential link between lysosomal-related genes and the occurrence and development of liver cancer. Understanding the molecular mechanisms of CLN3, GBA, and LAPTM4B in liver cancer cells may help to develop new therapeutic targets for liver cancer. In addition, Cathepsin A (CTSA), a lysosome-encapsulated cellular proteases, its abnormal expression promotes tumor growth and metastasis^23,24^^.^ Zhao et al.^47^ also reported that CTSA was overexpressed and associated with the carcinogenesis of liver cancer. BSG also known as Basigin or CD147, EMMPRIN, an immunoglobulin member, which can interact with extracellular, intracellular and membrane proteins and is the first reported protein to promote cancer development^48^. A recent study using CD147-CAR immunotherapy to treat HCC based on the abnormal high expression of CD47 and its negative correlation with prognosis^49^. Apelin (APLN) encodes an adipokine prepropeptide. Muto et al.^50^ disclosed that APLN overexpression was associated with arteriogenesis in HCC. In addition, through activation of the PI3K/Akt pathway, APLN regulates the progression of HCC^51^. As a lipid metabolism regulatory gene, Sort1 participated in the LDL metabolism and largely involved in the directional transport of various proteins in lysosomes^52,53^. Recent study reported that Sort1 exerted its function as pro-oncogenic molecules in HCC^54^. Studies have found that Annexin A2 (anx2) is related to tumor migration, epithelial mesenchymal transformation (EMT) and promotes tumor progression^55^. As the previous report, lysosome is significantly associated with cancer cell proliferation, invasion, metastasis, and gene expression regulation^56^.Consistent with the aforementioned reports, our results indicate that these 8 gene signatures are closely associated with malignant clinical features and immune therapy resistance in liver cancer. Furthermore, our results also suggest that these 8 gene signatures can independently predict overall survival outcome apart from known clinical and pathological risk factors. Additionally, we observed that all 8 model genes play a crucial role in the progression and development of tumors through the regulation of lysosomal-related pathways. Recently, a prognostic model of related lysosome-related genes has also been reported^41^. The authors used 8 genes (RAMP3, GPLD1, FABP5, CD68, CSPG4, SORT1, CSPG5, CSF3R) to construct a risk model, and the study showed that the risk model could better predict the clinical outcome, and the higher the risk, the worse the clinical outcome. In addition, the authors found significant differences in biological function, immune microenvironment, immunotherapy responsiveness and drug sensitivity between high-risk group and low-risk group. In terms of research content and conclusion, our study and the above study focus on lysosome-related genes and their relationship with hepatocellular carcinoma (HCC), with the purpose of identifying prognostic markers and evaluating their potential impact on the diagnosis, prognosis and treatment of HCC. At the same time, both studies found significant differences in clinical outcomes between high-risk and low-risk groups, that is, the high-risk group had worse clinical outcomes than the low-risk group, indicating the potential utility of the identified genetic signatures as prognostic markers. However, there are several differences between the two studies. First, there are differences in the specific lysosomal-associated genes that were identified as significant and used in risk modeling between the two studies, which may be related to the different gene sets that were included in the analyses. Second, the former study evaluated the differences in biological function, immune microenvironment, and drug sensitivity between high and low risk groups. However, our study evaluated tumor stemness, heterogeneity, genomic alteration status, immune-cell infiltration, and response to immunotherapy and chemotherapy. In addition, our study more comprehensively evaluated the early diagnostic value of risk models. Therefore, although the conclusions of the two studies are similar, there are still many differences in the overall research content and methods.It may be of great significance to further optimize and merge the research methods and research contents of the two studies.

Currently, AFP is still the most commonly used non-invasive diagnostic marker for HCC, but its diagnostic sensitivity and specificity are still relatively low^57^. It is noteworthy that the performance of our 8-gene signature in distinguishing HCC patients from normal samples and early-stage liver cancer is superior to AFP.. In this study, HCC patients with high LRRGs scores appear to be more sensitive to common clinical chemotherapy drugs, such as Sorafenib, Paclitaxel, Gemcitabine, and 5-Fluorouracil for liver cancer, illustrating that LRRS maybe a potentially tool for drug sensitivity prediction.In addition, considering the impact of LRRS on the clinical outcomes, a nomogram including LRRS, clinical features was constructed,which had a excellent predictive value for HCC. Therefore, the LRRGs signature constructed in this study may be a promising biomarker for the diagnosis and prognosis of HCC.

It has been reported that the number of lysosomes in the lysosomal network affects cell growth by activating mTOR protein^58^. When the number of lysosomes increases, the mTOR molecule on the surface of the lysosomal body becomes hyperactive. The GO, KEGG, and GSEA analysis may explain the causes of prognostic differences between the LRRS-classified HCC groups. Multiple immune-related pathways were also found to be enriched in low-LRRS group, such as T/B cell receptor signaling pathways and leukocyte transendothelial migration. It indicated that low-LRRS patients with higher immune activity might have a better prognosis. Immune cell infiltration is an indirect manifestation of immune activity. It has been reported that CD8 + T cells can induce anti-tumor response by producing interferon-(IFN)^59^. Th17 cells are considered to have high and long-term efficacy antitumor activity^60,61^. Interestingly, Treg cells can suppress immune activation by secreting immunosuppressive factors or expressing co inhibitory molecules^62^. As mentioned earlier, lysosomes are critically involved in tumor immunity. In our study, exhausted T and nTreg infiltrated high-LRRS group more than low-LRRS group. On the contrary, CD8-naive, Th17 cells, and Monocyte infiltrated low-LRRS group more than high-LRRS group. All these result suggestted that high-LRRS may relate to immunosuppression, and associate with a poor prognosis,while low-LRRS maybe relate to immune activity and achieve a well prognosis. Nowadays, a series of targeting drugs have been developed for HCC such as, anti-PD-1, anti-PD-L1 and anti-CTLA-4^63^. However, some success has been reported with immunotherapy in the treatment of HCC, the number of people who benefited from immunotherapy is still very low. Therefore, pre-treatment evaluation is particularly necessary. Our study showed a positive correlation between LRRS and TME scores, which provides a possibility for the prediction of immunotherapy. Predictably, TIDE scores did differ between the two LRRS groups and patients with low LRRS were more sensitive to immunotherapy, which is highly consistent with our analysis. It is further confirmed that LRRS still has potential value in predicting the efficacy of in tumor ICI therapy.

Tumor heterogeneity and stemness are strongly associated with the choice of cancer treatment and the length of overall survival time^64^. Our study revealed that LRRS was positively related to the tumor stemness and tumor heterogeneity, which mean that HCC cells with higher LRRS are more primitive and less differentiated. TMB is an important marker for predicting cancer efficacy, especially for immunotherapy^65^.Previous study reported that thymic epithelial tumors patients with high TMB had a significantly poor prognosis. We found no difference in TMB between the two groups, but regarding somatic mutation, significantly higher population mutation rate were observed in patients with high-LRRS, which may also indicate the poor prognosis in the high-LRRS group.

In summary, our study systematically analyzed and obtained the potential clinical value of lysosomal-related genes in HCC. Firstly, we revealed the aberrant expression profiles of lysosomal-related genes in HCC, confirming their pro-cancer role in HCC. Secondly, we constructed a lysosomal-related gene signature consisting of CLN3, GBA, CTSA, BSG, APLN, SORT1, ANXA2, and LAPTM4B, which demonstrated high performance in the diagnosis and prognosis of HCC patients. Additionally, this LRRGs signature was strongly associated with clinical features of malignant tumors, immune-suppressive tumor microenvironments, and chemotherapy response. Finally, the specific expression of CLN3, GBA, and LAPTM4B in Hepatocytes suggested their potential as biological markers for liver cells. In conclusion, the systematic evaluation of lysosomal-related genes in HCC can provide theoretical basis for their clinical application, help us understand the occurrence of liver cancer, and accelerate the development of new intervention strategies. However, our study also has some limitations. Firstly, although the results have been validated through multiple approaches, further clinical multicenter validation is still needed. Secondly, the specific mechanisms and roles of CLN3, GBA, and LAPTM4B in liver cells require further investigation. Thirdly, the potential mechanisms of lysosomes in chemotherapy response and immune-suppressive tumor microenvironment need further exploration.

### Supplementary Information

Supplementary Information.

Supplementary Table S1.

## Data Availability

The datasets used and/or analyzed during the current study are available from The Cancer Genome Atlas(TCGA, https://portal.gdc.cancer.gov/repository)(search term: TCGA-LIHC), International Cancer Genome Consortium (ICGC) Japanese liver cancer (ICGC-LIRI-JP) cohort (https://dcc.icgc.org/projects/LIRI-JP), HPA (http://www.proteinatlas.org), and from NCBI GEO: GSE144269 and GSE76427, as well as supplementary materials provided in the article.
