# Supplementary material for: Construction and validation of a novel lysosomal signature for hepatocellular carcinoma prognosis, diagnosis, and therapeutic decision-making
Source: Sci Rep. 2023 Dec 18;13:22624. doi: 10.1038/s41598-023-49985-3 (PMC10730614; doi:10.1038/s41598-023-49985-3)

Applied Biosystems

GeneMapper 4.0

CellLineAuthentication-HL-7702[L-02]

Sample File	Sample Name	Panel	OS	SQ
58_B08_WeiWeiXingXiaoShi1-6XBJD7-0607.fsa	HL-7702[L-02]	STR Profile 1-human		

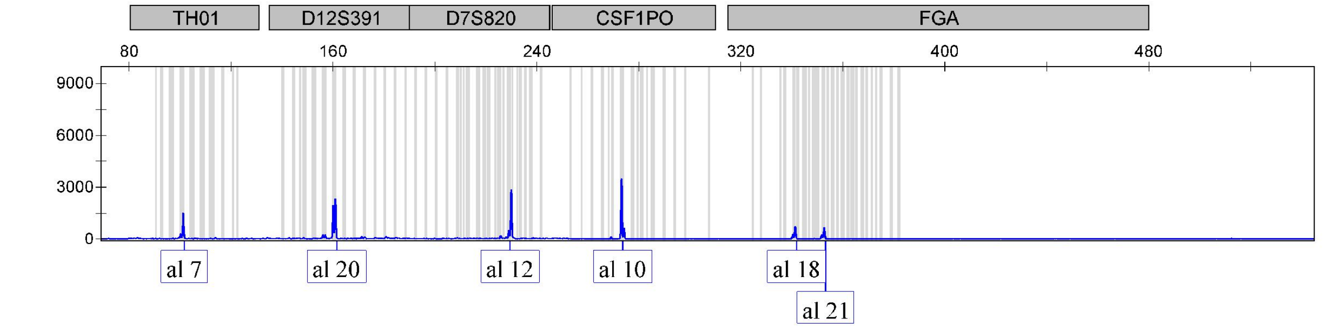

58_B08_WeiWeiXingXiaoShi1-6XBJD7-0607.fsa	HL-7702[L-02]	STR Profile 1-human		

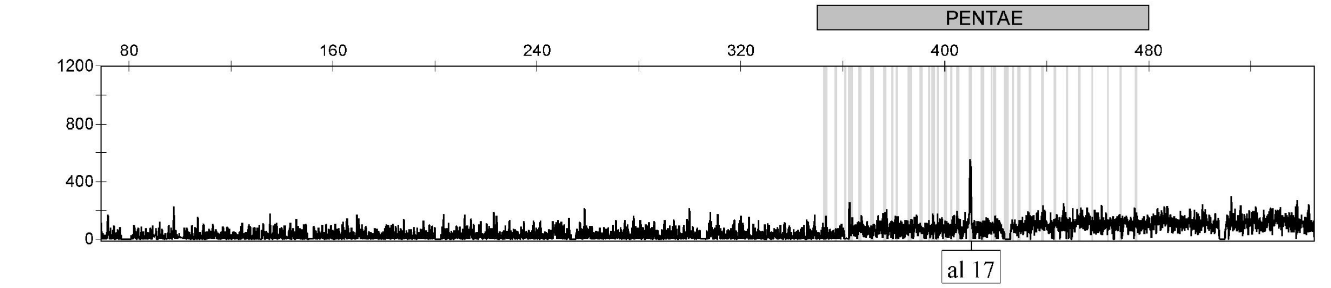

61_E08_WeiWeiXingXiaoShi1-6XBJD7-0607.fsa	HL-7702[L-02]	STR Profile 2-human		

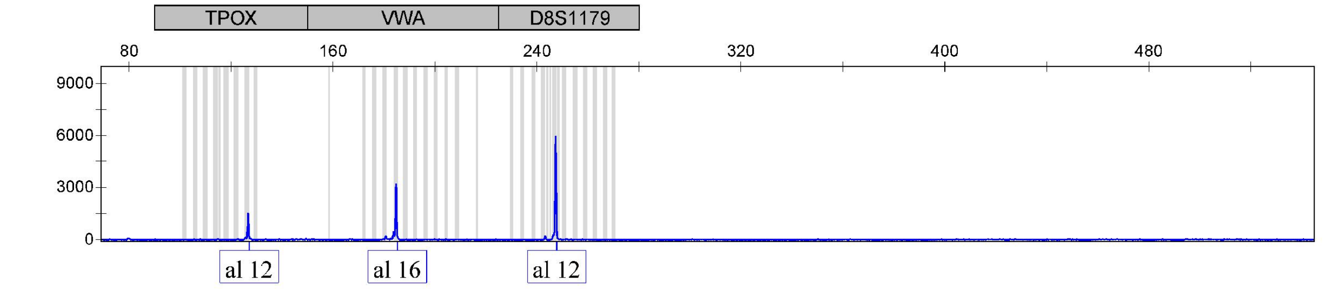

61_E08_WeiWeiXingXiaoShi1-6XBJD7-0607.fsa	HL-7702[L-02]	STR Profile 2-human		

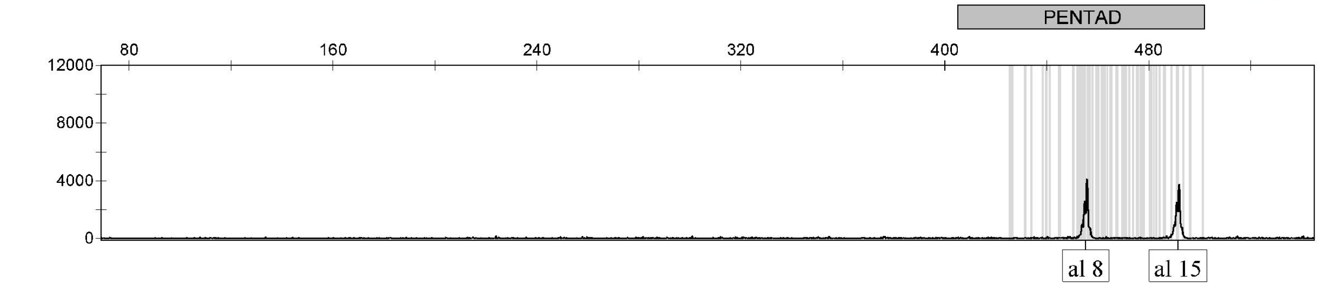

GeneMapper 4.0

CellLineAuthentication-HL-7702[L-02]

Sample File	Sample Name	Panel	OS	SQ
21_E03_CellLineAuthentication-0608.fsa	HL-7702[L-02]	STR Profile 3-human		

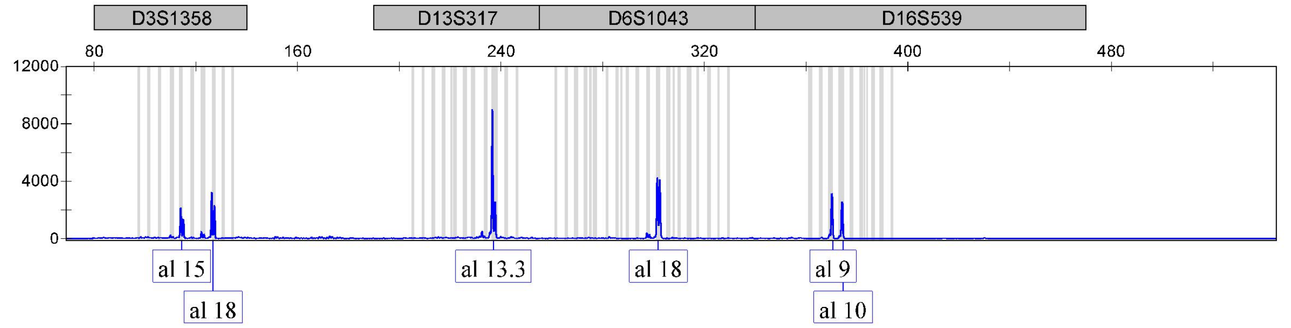

Sample File	Sample Name	Panel	OS	SQ
21_E03_CellLineAuthentication-0608.fsa	HL-7702[L-02]	STR Profile 3-human		

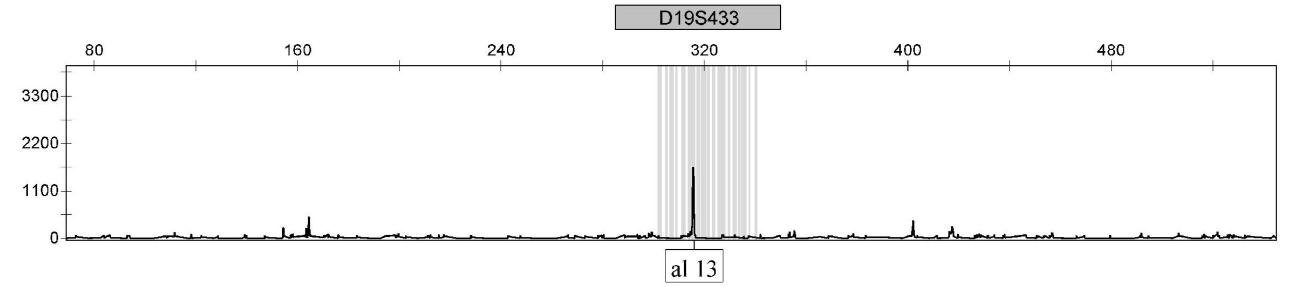

Sample File	Sample Name	Panel	OS	SQ
18_B03_CellLineAuthentication-0608.fsa	HL-7702[L-02]	STR Profile 4-human		

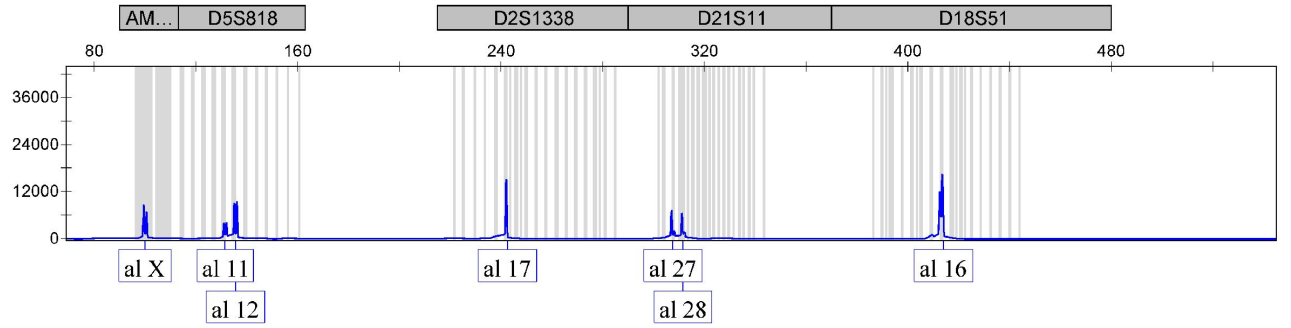

Sample File	Sample Name	Panel	OS	SQ
18_B03_CellLineAuthentication-0608.fsa	HL-7702[L-02]	STR Profile 4-human		

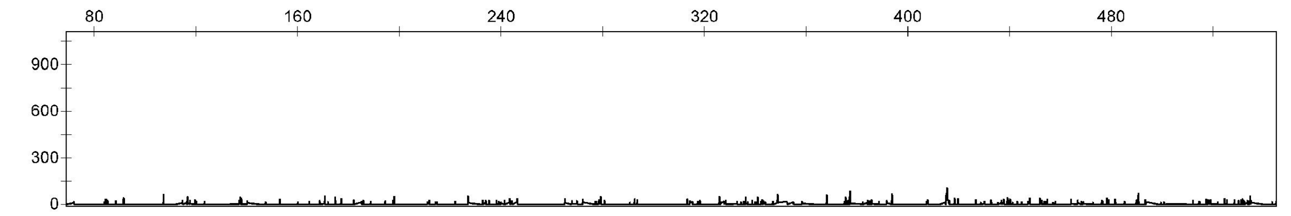

Supplement: Supplementary file 1 — Supplementary Information. [file 41598_2023_49985_MOESM1_ESM.zip › Supplementary S2/lo2-STR/HL-7702[L-02]细胞图谱.pdf]
