# Supplementary material for: Construction and validation of a novel lysosomal signature for hepatocellular carcinoma prognosis, diagnosis, and therapeutic decision-making
Source: Sci Rep. 2023 Dec 18;13:22624. doi: 10.1038/s41598-023-49985-3 (PMC10730614; doi:10.1038/s41598-023-49985-3)
Supplement: Supplementary file 1 — Supplementary Information. [file 41598_2023_49985_MOESM1_ESM.zip › Supplementary S2/lo2-STR/HL-7702[L-02]细胞鉴定报告.pdf]

细胞遗传质量鉴定检测

Cell Line Authentication Service

STR 基因型检测报告

送检单位：上海弘顺生物科技有限公司

检品名称：细胞系

报告日期：2017-06-12

样 品 信 息

样品编号：

客户样本编号	公司编号
HL-7702[L-02]	20170602-01

样品数量： 1

样品性状： 细胞系

检测项目： STR

送检单位： 上海弘顺生物科技有限公司

检测方法： 用 Axygen 的基因组抽提试剂盒提取 DNA，采用 20- STR 扩增方案扩增，在 ABI 3730XL 型遗传分析仪上对 STR 位点和性别基因 Amelogenin 进行检测。

检 测 结 果

(一)　检验基本情况

	多等位基因	匹配细胞系	细胞库	EV 值	匹配说明
20170602-01	无		DSMZ		无

样本基因型检验结果

- 多等位基因指三等位及以上基因现象。
- 本次检测各细胞分型结果良好。

(二)　各样本描述

- 20170602-01：该株细胞 DNA 分型在细胞系检索中**没有找到匹配**的细胞系。本次检测在该细胞系中**没有发现多等位基因**。（该细胞系未发现发现多等位基因、未发现交叉污染，细胞系本身无异常，因数据库未登录相 STR 信息，无法匹配，若为发表论文计可提交该数据给杂志即可）

备注：待测细胞系与收录于 ATCC, DSMZ, JCRB 和 RIKEN 数据库的细胞系 STR 数据进行比对，未收录于以上细胞库的细胞系将无法匹配。

（三）样本分型结果

细胞 20170602-01 的 STR 位点和 Amelogenin 位点的基因分型结果						
Loci	送检细胞 STR 信息			细胞库细胞 STR 信息		
	送检细胞名：HL-7702[L-02]			细胞库细胞名：		
	Allele1	Allele2	Allele3	Allele1	Allele2	Allele3
D5S818	11	12				
D13S317	13.3	13.3				
D7S820	12	12				
D16S539	9	10				
VWA	16	16				
TH01	7	7				
AMEL	X	X				
TPOX	12	12				
CSF1PO	10	10				
D12S391	20	20				
FGA	18	21				
D2S1338	17	17				
D21S11	27	28				
D18S51	16	16				
D8S1179	12	12				
D3S1358	15	18				
D6S1043	18	18				
PENTAE	7	17				
D19S433	13	13				
PENTAD	8	15				

其 他 说 明

(一)　分型方案及位点分布

	方案 1	方案 2	方案 3	方案 4
1	TH01	TPOX	D3S1358	AMEL
2	D12S391	VWA	D13S317	D5S818
3	D7S820	D8S1179	D6S1043	D2S1338
4	CSF1PO	PENTAD	D16S539	D21S11
5	FGA		D19S433	D18S51
6	PENTAE			

实验方案及位点

(二)　STR 数据库比对

本公司采用 DSMZ tools 进行细胞系比对，其中包含来自于 ATCC, DSMZ, JCRB 和 RIKEN 数据库的 2455 个细胞系 STR 数据。如果待检测细胞未收录于以上细胞库或这是自行建立的新细胞系将无法进行比对，用户需根据细胞分型结果自行与其他数据库进行比对。

主要实验人员： 刘志新

复核人: 宣程

负责人： 张晨曦

上海弘顺生物科技有限公司

签发日期： 2017 年 06 月 12 日
